# Two Highly Efficient Prime Editing Systems Based on the Csy4 CRISPR Endonuclease

**DOI:** 10.1111/pbi.70337

**Published:** 2025-09-24

**Authors:** Yu Lu, Dexin Qiao, Junya Wang, Wei Sun, Zhenghong Cao, Minhui Lu, Yiping Chai, Yuanyuan Jiang, Cuiping Xin, Xiaohan Liu, Siyun Li, Syeda Leeda Gul, Qi‐Jun Chen

**Affiliations:** ^1^ State Key Laboratory of Plant Environmental Resilience, College of Biological Sciences China Agricultural University Beijing China; ^2^ Center for Crop Functional Genomics and Molecular Breeding China Agricultural University Beijing China

**Keywords:** Cas6f, Csy4, prime editing, rice, split prime editor

Dear Editor,

Prime editing is an advanced CRISPR/Cas‐derived technology designed to enable precise genetic modifications, including base substitutions, insertions and deletions, at targeted genomic loci (Anzalone et al. 2019). Compared to the unsplit prime editor (PE) system, split PE strategies—such as split inteins, MS2, SunTag, CC‐PE and direct split PEs with untethered reverse transcriptase (RT)—offer a more adaptable and efficient approach for size‐constrained delivery systems, such as viral delivery, and facilitate the ongoing development of new PEs based on various CRISPR/Cas systems and RTs (Grunewald et al. 2023; Liu et al. 2022; Mu et al. 2024; Wei et al. 2025). Csy4 (also known as Cas6f) is the key enzyme responsible for crRNA production in CRISPR subtype I‐F (Sternberg et al. 2012). Csy4 binds with equal affinity to both its substrate pre‐crRNA and product crRNA (Sternberg et al. 2012). The Csy4 system has been effectively employed in prime editing for processing pegRNAs (Liu et al. 2021; Ni et al. 2023).

In this report, we hypothesised that the Csy4 system could be leveraged to develop a new split PE (sPE) (Figure 1a). Csy4's strong and persistent binding to the Csy4 recognition sequence (Csy4RS), even after cleavage, allows the formation of a functional epegRNA‐Csy4RS‐Csy4 complex (Figure 1a). By fusing RT to Csy4, this complex (epegRNA‐Csy4RS‐Csy4‐RT) can be captured by the SpCas9KK‐H840A nickase to drive prime editing, thereby creating a new sPE (Figure 1a). Based on these rationales, we generated two new sPEs (9s42: Cas9n + Csy4‐RT2 and 9s24: Cas9n + RT2‐Csy4), along with a sPE control with untethered RT (9s2) (Figure 1a). To assess Csy4's activity of Csy4‐fused proteins, we generated two fusion PEs of Csy4 and PEmax (492: Csy4‐PEmax and 924: PEmax‐Csy4), and two Csy4‐based or non‐Csy4‐based unsplit PE controls, 4A92 (Csy4‐P2A‐PEmax) and 92 (PEmax) (Figure 1a). For non‐Csy4‐based PEs, we generated an RNA expression system, 2 × PE3V1, by combining a composite promoter with two U3 promoters to separately drive epegRNA and sgRNA expression (Figure 1b). For Csy4‐based PEs, we placed the 20‐bp Csy4RS at both ends of epegRNAs and sgRNAs, producing 2 × PE3RS cassette (Figure 1b). To test whether evopreQ1 (Chen et al. 2021) could be removed from pegRNAs without compromising the editing efficiency of Csy4‐based PEs, we generated evopreQ1‐free Csy4RS‐containing RNA cassette (2 × PE3RS∆E). Thus, we generated a total of eight types of duplex PEs by combining the seven split or unsplit PE proteins with three RNA cassettes (Figure 1b,c; Methods S1).

**FIGURE 1:**
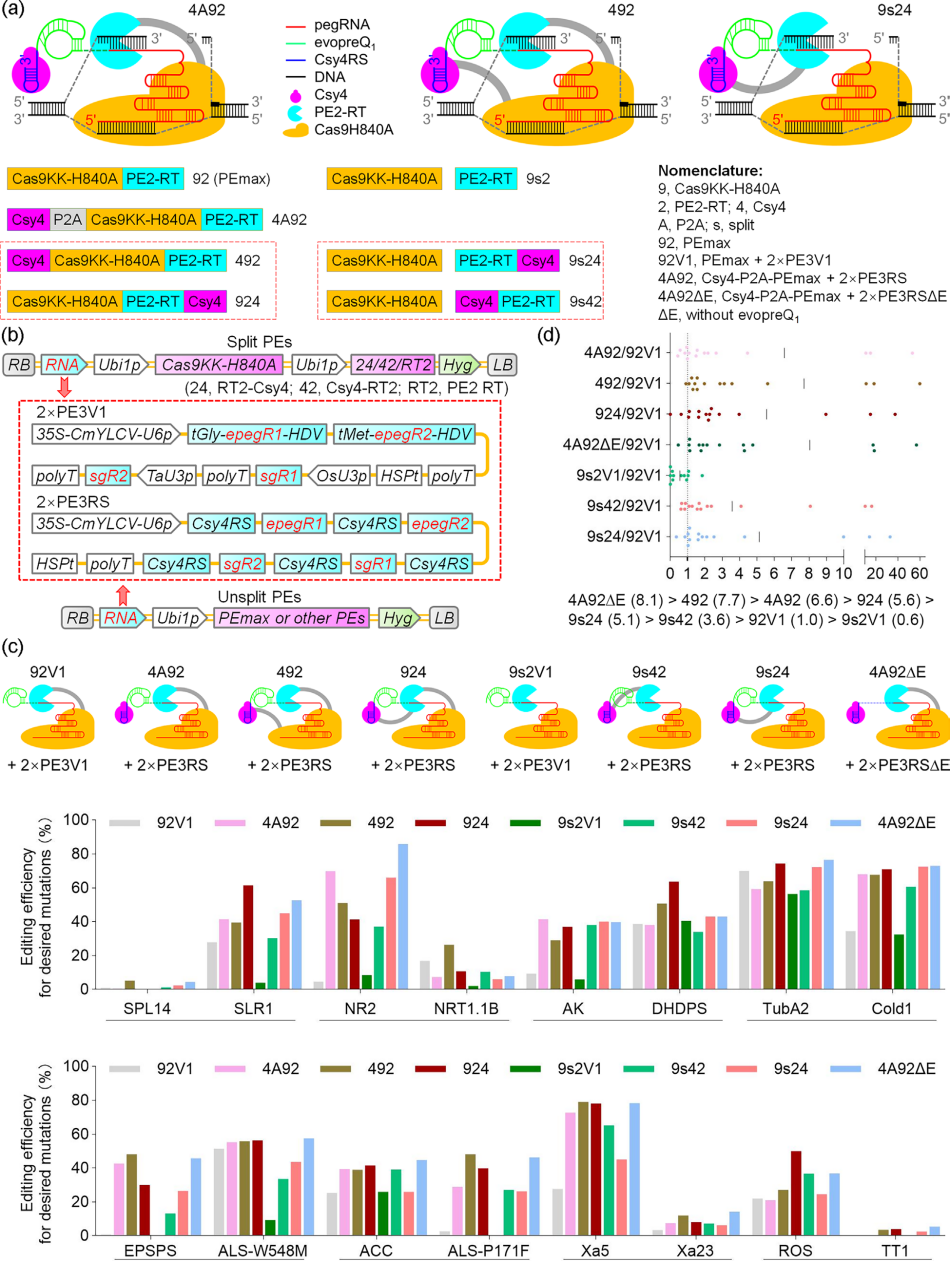
The Csy4 system enabled the creation of a new split prime editor. (a) Schematic diagram of three types of Csy4‐based prime editors, with the type 4A92 reported previously and two new types described in this paper. Structures of seven types of prime editor proteins tested in this study are indicated. (b) Schematic representation of the T‐DNA constructs used in the generation of transgenic rice, involving eight types of duplex PEs. (c) Comparative analysis of editing efficiencies of eight types of duplex PEs across 12 or 16 targets from 11 or 15 genes for 9s2V1 or the other PEs, respectively, in transgenic rice. Schematic diagram of the eight types of prime editors is indicated. (d) Average fold change in prime editing efficiency at all 12 or 16 target sites, except TT1, in rice.

We compared editing efficiencies of the eight PEs across 12 or 16 targets from 11 or 15 genes in transgenic rice plants (Figure 1c, Figure S1; Table S1). Our results showed that all six Csy4‐based PEs, including the two sPEs, substantially improved overall editing efficiencies compared to the non‐Csy4‐based unsplit PE control (92V1) and the sPE control with untethered RT (9s2V1) (Figure 1c). The fold improvements relative to the 92V1 control were 6.58 (4A92), 7.72 (492), 5.56 (924), 0.56 (9s2V1), 3.57 (9s42), 5.13 (9s24) and 8.05 (4A92∆E) (Figure 1d). Although the two sPEs showed slightly lower overall editing efficiencies than the other Csy4‐based PEs, they still performed much better than the non‐Csy4‐based control 92 V1. In contrast, the sPE with untethered RT (9s2V1) showed lower overall editing efficiency than the control PE (92V1). The observed lower editing efficiency of the Csy4‐based sPEs, as compared to unsplit Csy4‐based PEs, may be attributed to the slower recruitment of pegRNA‐Csy4RS‐Csy4‐RT to Cas9 as compared to the pegRNA‐Csy4RS‐Csy4 complex. The Csy4‐based sPE system has the unique advantage of coupling pegRNA processing and RT recruitment into a single, streamlined step, eliminating the need for additional RT recruitment. In contrast, other sPEs require two distinct recruitment steps. For example, intein‐based sPEs necessitate a first step for trans‐splicing to reconstruct the PE protein, followed by a second step to recruit pegRNAs. Similarly, MS2‐PE, SunTag‐PE and CC‐PE involve two independent steps for the recruitment of pegRNAs and RT to the SpCas9 nickase or target site (Grunewald et al. 2023; Liu et al. 2022; Mu et al. 2024; Wei et al. 2025).

Notably, the uncleavable Csy4‐PEmax fusion (492) achieved higher overall editing efficiency than the cleavable Csy4‐P2A‐PEmax fusion (4A92) (Figure 1c,d). The superior performance of Csy4‐PEmax can be attributed to its more effective recruitment of pegRNAs to PEmax compared to the standalone Csy4 and PEmax produced by the cleavage of Csy4‐P2A‐PEmax. In addition, our results confirmed that the deletion of evopreQ1 increased editing efficiency by 1.8‐fold (4A92∆E/4A92) (Figure 1c,d), suggesting that the pegRNA‐Csy4RS‐Csy4 complex could provide sufficient protection against degradation at the 3′ end of pegRNAs and reduce unwanted hybridisation between the spacer and the PBS. Consistent with these results, pairing PE7 with pegRNA‐polyU produced intended editing efficiencies similar to or higher than those from PE7 with pegRNA‐evopreQ1‐polyU (Yan et al. 2024). Thus, both Csy4‐based PE and PE7 systems leverage RNA‐binding proteins to stabilise pegRNAs (Yan et al. 2024). Given that Csy4 binds more specifically to pegRNA‐Csy4RS than La binds to pegRNA‐polyU, further investigation into whether Csy4‐based PE can outperform PE7 would be valuable.

In earlier experiments, Csy4 was shown to negatively affect maize and Arabidopsis transformation (Jiang et al. 2020); however, we did not observe such effects in this study. Since it was reported that the Csy4‐mediated cytotoxicity was observed when Csy4 plasmid concentrations were high (Nissim et al. 2014), we therefore hypothesise that the negative effects previously reported were due to the overly high Csy4 expression level, which was caused by the strong constitutive fusion promoter, 35S‐CmYLCV (Jiang et al. 2020).

In conclusion, we developed a new Csy4‐based split PE (SpCas9KK‐H840A + Csy4‐RT), characterised by its ability to couple pegRNA processing and protection with RT recruitment. Our results show that this split PE substantially improved editing efficiency compared to unsplit PE and split PE with untethered RT in rice plants. Additionally, we demonstrate that the non‐cleavable Csy4‐PEmax variant outperformed its cleavable counterpart, Csy4‐P2A‐PEmax. Collectively, these findings provide a strong foundation for applying Csy4‐based split PEs and the non‐cleavable Csy4‐PE in both agriculture and gene therapy and also facilitate building a versatile platform for the ongoing development of new PEs based on various CRISPR/Cas systems and RTs.

## Author Contributions

Q.‐J.C. conceived and designed the research. Y.L., D.Q., J.W., M.L., Y.C., Y.J., C.X., X.L., S.L. and S.L.G. conducted the experiments. Y.L., D.Q., J.W., W.S. and Z.C. analysed the data. Q.‐J.C., Y.L., D.Q. and J.W. wrote the manuscript.

## Supporting information


**Figure S1:** Sorting‐based editing efficiencies of eight types of duplex PEs.
**Table S1:** Mutation efficiencies of eight types of PEs.
**Methods S1**. Vector construction, prime editing analysis and so on.

## Data Availability

The data that support the findings of this study are available on request from the corresponding author. The data are not publicly available due to privacy or ethical restrictions.
