# Supplementary material for: Two Highly Efficient Prime Editing Systems Based on the Csy4 CRISPR Endonuclease
Source: Plant Biotechnol J. 2025 Sep 24;24(2):828–30. doi: 10.1111/pbi.70337 (PMC12906836; doi:10.1111/pbi.70337)
Supplement: Supplementary file 1 — Figure S1: Sorting‐based editing efficiencies of eight types of duplex PEs. Table S1: Mutation efficiencies of eight types of PEs. Methods S1. Vector construction, prime editing analysis and so on. [file PBI-24-828-s001.pdf]

Supporting information

Two highly efficient prime editing systems based on the Csy4 CRISPR endonuclease

Yu **Lu**[1,†], Dexin **Qiao**[1,†], Junya **Wang**[1,†], Wei **Sun**[1], Zhenghong **Cao**[1], Minhui **Lu**[2], Yiping **Chai**[1], Yuanyuan **Jiang**[1], Cuiping **Xin**[1], Xiaohan **Liu**[1], Siyun **Li**[1], Syeda Leeda **Gul**[1], Qi-Jun **Chen**[1,2*]

[1]State Key Laboratory of Plant Environmental Resilience, College of Biological Sciences, China Agricultural University, Beijing 100193, China

[2]Center for Crop Functional Genomics and Molecular Breeding, China Agricultural University, Beijing 100193, China

[†]These authors contributed equally to this article.

[*]Correspondence: Qi-Jun Chen (qjchen@cau.edu.cn)

Table of contents

Figure S1 2
Table S1 4
Methods S1 9
Vector construction 9
Rice transformation and prime editing analysis 10
Supplemental Sequences 11
Supplemental References 30

Figure S1

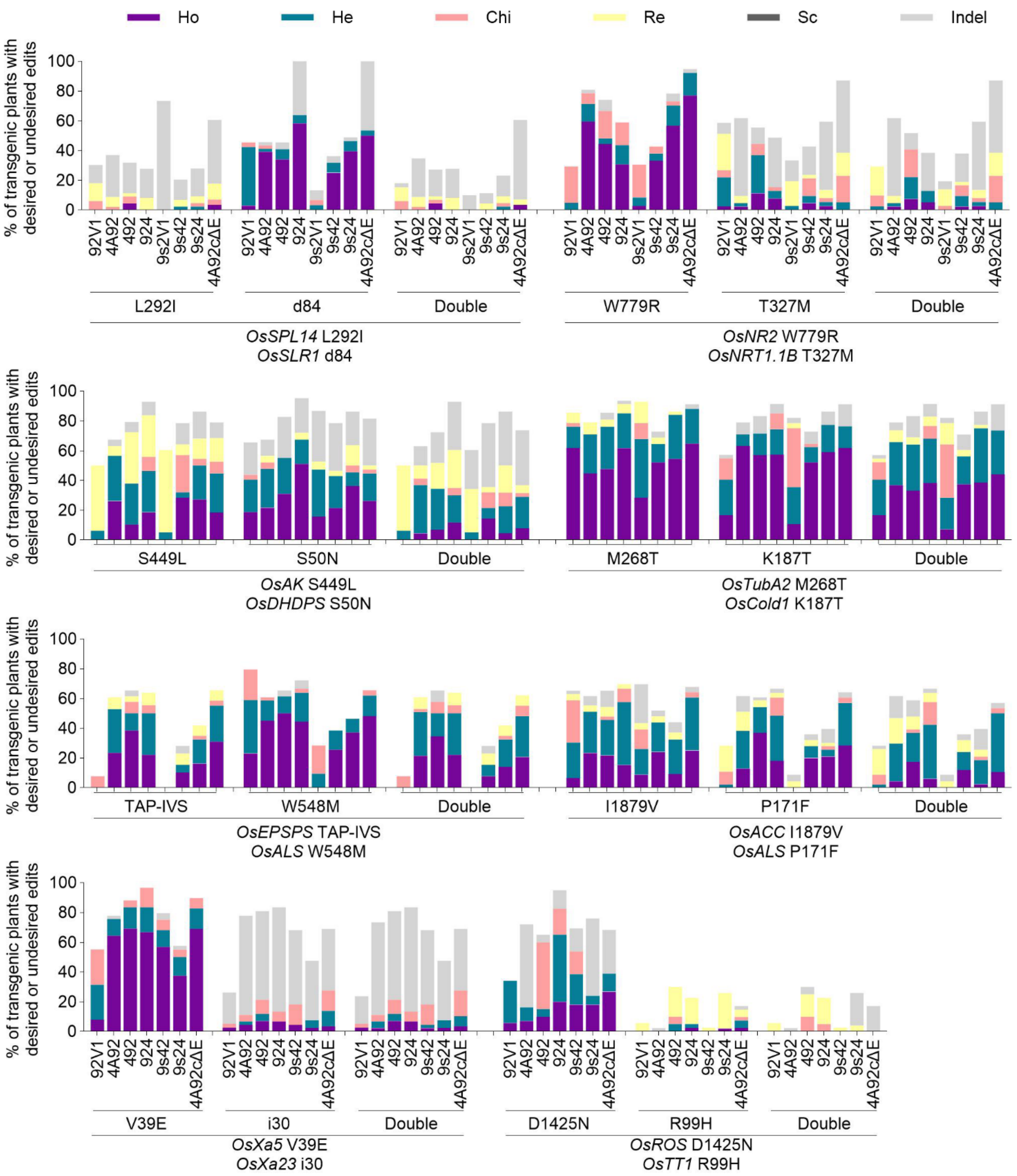

Figure S1 Sorting-based editing efficiencies of eight types of duplex PEs.

Ho, He, and Chi are homozygous, heterozygous, and chimeric mutant lines, respectively. Re, DNA repair-derived byproducts with only some of the target bases edited when installing multiple-base substitution edits. Sc, pegRNA scaffold-derived byproducts. The transgenic

plants were analyzed by deep sequencing of PCR amplicons with 5% threshold. Lines with 100% deep-sequencing reads representing only one type of mutation with desired edits were scored as homozygous. Non-homozygous lines containing more than 45% deep sequencing reads representing desired edits were scored as heterozygous. Non-homozygous and non-heterozygous mutant lines were scored as Chi, Re, Sc, or Indel lines when the main mutation types were desired edits, Re byproducts, Sc byproducts, or Indel byproducts, respectively. The sorting-based mutation efficiency was calculated based on the ratio of the number of mutants to the total number of transgenic plants.

92V1, PEmax + 2×PE3V1; 4A92, Csy4-P2A-PEmax + 2×PE3RS; 492, Csy4-PEmax + 2×PE3RS; 924, PEmax-Csy4 + 2×PE3RS; 9s2V1, Cas9KK-H840A + RT2 + 2×PE3V1; 9s42, Cas9KK-H840A + Csy4-RT2 + 2×PE3RS; 9s24, Cas9KK-H840A + RT2-Csy4 + 2×PE3RS; 4A92ΔE, Csy4-P2A-PEmax + 2×PE3RSΔE; RT2, PE2-RT.

Table S1

Table S1 Mutation efficiencies of eight types of PEs

Target	PEs	No. of lines	Gene	Average percentage of desired reads (%)	Ratio of prime-edited lines (%)					
					Ho	He	Chi	Re	Indel	Sc
SPL14 & SLR1	92V1	33	SPL14	0.9	0.0	0.0	6.1	12.1	12.1	0.0
			SLR1	28.0	3.0	39.4	3.0	0.0	0.0	0.0
			Double	—	0.0	0.0	6.1	9.1	3.0	0.0
	4A92	46	SPL14	0.5	0.0	0.0	2.2	6.5	28.3	0.0
			SLR1	41.4	39.1	2.2	2.2	0.0	2.2	0.0
			Double	—	0.0	0.0	2.2	6.5	26.1	0.0
	492	44	SPL14	5.2	4.5	0.0	4.5	2.3	20.5	0.0
			SLR1	39.4	34.1	6.8	0.0	0.0	4.5	0.0
			Double	—	4.5	0.0	2.3	2.3	18.2	0.0
	924	36	SPL14	0.0	0.0	0.0	0.0	8.3	19.4	0.0
			SLR1	61.6	58.3	5.6	0.0	0.0	36.1	0.0
			Double	—	0.0	0.0	0.0	8.3	19.4	0.0
	9s2V1	30	SPL14	0.0	0.0	0.0	0.0	0.0	73.3	0.0
			SLR1	3.9	0.0	3.3	3.3	0.0	6.7	0.0
			Double	—	0.0	0.0	0.0	0.0	10.0	0.0
	9s42	44	SPL14	1.2	0.0	2.3	0.0	4.5	13.6	0.0
			SLR1	30.3	25.0	6.8	0.0	0.0	4.5	0.0
			Double	—	0.0	0.0	0.0	4.5	6.8	0.0
	9s24	43	SPL14	2.3	0.0	2.3	2.3	4.7	18.6	0.0
			SLR1	45.0	39.5	7.0	0.0	0.0	2.3	0.0
			Double	—	0.0	2.3	2.3	4.7	14.0	0.0
	4A92cΔE	28	SPL14	4.4	3.6	0.0	3.6	10.7	42.9	0.0
			SLR1	52.5	50.0	3.6	0.0	0.0	46.4	0.0
			Double	—	3.6	0.0	0.0	3.6	53.6	0.0
NR2 & NRT1.1b	92V1	41	NR2	4.6	0.0	4.9	24.4	0.0	0.0	0.0
			NRT1.1B	16.9	2.4	19.5	4.9	24.4	7.3	0.0
			Double	—	0.0	2.4	7.3	19.5	0.0	0.0
	4A92	42	NR2	69.9	59.5	11.9	7.1	0.0	2.4	0.0
			NRT1.1B	7.2	2.4	2.4	0.0	4.8	52.4	0.0
			Double	—	2.4	2.4	0.0	4.8	52.4	0.0
	492	27	NR2	51.1	44.4	3.7	18.5	0.0	7.4	0.0
			NRT1.1B	26.4	11.1	25.9	7.4	0.0	11.1	0.0
			Double	—	7.4	14.8	18.5	0.0	11.1	0.0
	924	39	NR2	41.3	30.8	12.8	15.4	0.0	0.0	0.0
			NRT1.1B	10.6	7.7	5.1	2.6	0.0	33.3	0.0
			Double	—	5.1	7.7	0.0	0.0	25.6	0.0
	9s2V1	36	NR2	8.5	2.8	5.6	22.2	0.0	0.0	0.0
			NRT1.1B	2.0	0.0	2.8	0.0	16.7	13.9	0.0
			Double	—	0.0	0.0	2.8	11.1	5.6	0.0

Target	PEs	No. of lines	Gene	Average percentage of desired reads (%)	Ratio of prime-edited lines (%)					
					Ho	He	Chi	Re	Indel	Sc
	9s42	42	NR2	37.1	33.3	4.8	4.8	0.0	0.0	0.0
			NRT1.1B	10.4	4.8	4.8	11.9	2.4	19.0	0.0
			Double	—	2.4	7.1	7.1	2.4	19.0	0.0
	9s24	37	NR2	65.9	56.8	13.5	2.7	0.0	5.4	0.0
			NRT1.1B	5.5	2.7	2.7	2.7	5.4	45.9	0.0
			Double	—	2.7	2.7	2.7	5.4	45.9	0.0
	4A92cΔE	39	NR2	85.8	76.9	15.4	0.0	0.0	2.6	0.0
			NRT1.1B	7.8	0.0	5.1	17.9	15.4	48.7	0.0
			Double	—	0.0	5.1	17.9	15.4	48.7	0.0
AK & DHDPS	92V1	32	AK	9.3	0.0	6.3	0.0	43.8	0.0	0.0
			DHDPS	38.6	18.8	21.9	3.1	0.0	21.9	0.0
			Double	—	0.0	6.3	0.0	43.8	0.0	0.0
	4A92	46	AK	41.5	26.1	30.4	0.0	6.5	4.3	0.0
			DHDPS	38.1	21.7	26.1	4.3	4.3	10.9	0.0
			Double	—	4.3	32.6	4.3	8.7	13.0	0.0
	492	29	AK	29.3	10.3	27.6	0.0	34.5	6.9	0.0
			DHDPS	50.7	31.0	24.1	0.0	0.0	27.6	0.0
			Double	—	6.9	27.6	0.0	17.2	20.7	0.0
	924	43	AK	37.2	18.6	27.9	9.3	27.9	9.3	0.0
			DHDPS	63.7	51.2	16.3	0.0	4.7	23.3	0.0
			Double	—	11.6	18.6	4.7	25.6	32.6	0.0
	9s2V1	38	AK	5.9	0.0	5.3	0.0	55.3	0.0	0.0
			DHDPS	40.5	15.8	31.6	0.0	5.3	34.2	0.0
			Double	—	0.0	5.3	0.0	28.9	26.3	0.0
	9s42	28	AK	37.7	28.6	3.6	25.0	7.1	14.3	0.0
			DHDPS	33.7	21.4	21.4	0.0	3.6	32.1	0.0
			Double	—	14.3	7.1	10.7	3.6	42.9	0.0
	9s24	22	AK	40.2	27.3	22.7	4.5	13.6	18.2	0.0
			DHDPS	42.8	36.4	9.1	4.5	13.6	22.7	0.0
			Double	—	4.5	18.2	9.1	18.2	36.4	0.0
	4A92cΔE	38	AK	39.8	18.4	26.3	7.9	15.8	10.5	0.0
			DHDPS	42.7	26.3	18.4	2.6	2.6	31.6	0.0
			Double	—	7.9	21.1	2.6	5.3	36.8	0.0
TubA2 & Cold1	92V1	42	TubA2	70.0	61.9	14.3	2.4	7.1	0.0	0.0
			Cold1	34.4	16.7	23.8	14.3	0.0	2.4	0.0
			Double	—	16.7	23.8	11.9	2.4	2.4	0.0
	4A92	38	TubA2	59.2	44.7	26.3	0.0	7.9	0.0	0.0
			Cold1	68.0	63.2	7.9	0.0	0.0	7.9	0.0
			Double	—	36.8	28.9	0.0	7.9	5.3	0.0
	492	42	TubA2	63.9	47.6	28.6	0.0	4.8	4.8	0.0
			Cold1	67.7	57.1	14.3	0.0	0.0	11.9	0.0
			Double	—	33.3	31.0	0.0	4.8	14.3	0.0

Target	PEs	No. of lines	Gene	Average percentage of desired reads (%)	Ratio of prime-edited lines (%)					
					Ho	He	Chi	Re	Indel	Sc
	924	47	TubA2	74.3	61.7	23.4	0.0	6.4	2.1	0.0
			Cold1	70.9	57.4	17.0	10.6	0.0	6.4	0.0
			Double	—	38.3	29.8	8.5	6.4	8.5	0.0
	9s2V1	28	TubA2	56.4	28.6	39.3	10.7	14.3	0.0	0.0
			Cold1	32.4	10.7	25.0	39.3	3.6	3.6	0.0
			Double	—	7.1	21.4	35.7	14.3	3.6	0.0
	9s42	48	TubA2	58.5	52.1	12.5	0.0	4.2	4.2	0.0
			Cold1	60.7	52.1	10.4	2.1	0.0	8.3	0.0
			Double	—	37.5	18.8	0.0	4.2	10.4	0.0
	9s24	44	TubA2	72.3	54.5	29.5	0.0	2.3	0.0	0.0
			Cold1	72.6	59.1	18.2	0.0	0.0	9.1	0.0
			Double	—	38.6	36.4	0.0	2.3	9.1	0.0
	4A92cΔE	34	TubA2	76.5	64.7	23.5	0.0	0.0	2.9	0.0
			Cold1	73.5	61.8	14.7	0.0	0.0	14.7	0.0
			Double	—	44.1	29.4	0.0	0.0	17.6	0.0
EPSPS & W548	92V1	39	EPSPS	0.8	0.0	0.0	7.7	0.0	0.0	0.0
			W548	51.4	23.1	35.9	20.5	0.0	0.0	0.0
			Double	—	0.0	0.0	7.7	0.0	0.0	0.0
	4A92	51	EPSPS	42.6	23.5	29.4	0.0	7.8	0.0	0.0
			W548	55.3	45.1	13.7	2.0	0.0	0.0	0.0
			Double	—	21.6	29.4	2.0	7.8	0.0	0.0
	492	26	EPSPS	48.1	38.5	11.5	7.7	3.8	3.8	0.0
			W548	55.9	50.0	11.5	0.0	0.0	3.8	0.0
			Double	—	34.6	15.4	7.7	0.0	7.7	0.0
	924	36	EPSPS	39.9	22.2	27.8	5.6	8.3	0.0	0.0
			W548	56.3	44.4	19.4	2.8	0.0	5.6	0.0
			Double	—	22.2	27.8	5.6	8.3	0.0	0.0
	9s2V1	21	EPSPS	0.0	0.0	0.0	0.0	0.0	0.0	0.0
			W549	9.2	0.0	9.5	19.0	0.0	0.0	0.0
			Double	—	0.0	0.0	0.0	0.0	0.0	0.0
	9s42	39	EPSPS	13.0	10.3	5.1	0.0	7.7	5.1	0.0
			W548	33.5	25.6	12.8	0.0	0.0	0.0	0.0
			Double	—	7.7	7.7	0.0	7.7	5.1	0.0
	9s24	43	EPSPS	26.5	16.3	16.3	2.3	7.0	0.0	0.0
			W548	43.6	37.2	9.3	0.0	0.0	0.0	0.0
			Double	—	14.0	18.6	2.3	7.0	0.0	0.0
	4A92cΔE	29	EPSPS	45.7	31.0	24.1	3.4	6.9	0.0	0.0
			W548	57.6	48.3	13.8	3.4	0.0	0.0	0.0
			Double	—	20.7	27.6	6.9	6.9	0.0	0.0
ACC & P171	92V1	46	ACC	25.2	6.5	23.9	28.3	4.3	2.2	0.0
			P171	2.6	0.0	2.2	8.7	17.4	0.0	0.0
			Double	—	0.0	2.2	6.5	17.4	2.2	0.0

Target	PEs	No. of lines	Gene	Average percentage of desired reads (%)	Ratio of prime-edited lines (%)					
					Ho	He	Chi	Re	Indel	Sc
	4A92	47	ACC	39.3	23.4	27.7	0.0	4.3	6.4	0.0
			P171	28.9	12.8	25.5	0.0	12.8	10.6	0.0
			Double	—	4.3	25.5	0.0	17.0	14.9	0.0
	492	46	ACC	38.9	21.7	23.9	2.2	6.5	10.9	0.0
			P171	48.1	37.0	17.4	0.0	4.3	2.2	0.0
			Double	—	17.4	19.6	2.2	8.7	10.9	0.0
	924	33	ACC	41.4	15.2	42.4	9.1	3.0	0.0	0.0
			P171	39.8	18.2	30.3	12.1	3.0	3.0	0.0
			Double	—	6.1	36.4	15.2	6.1	3.0	0.0
	9s2V1	23	ACC	26.0	8.7	17.4	13.0	4.3	26.1	0.0
			P171	0.0	0.0	0.0	0.0	4.3	4.3	0.0
			Double	—	0.0	0.0	0.0	4.3	4.3	0.0
	9s42	25	ACC	39.1	24.0	20.0	0.0	4.0	4.0	0.0
			P171	27.1	20.0	8.0	0.0	4.0	4.0	0.0
			Double	—	12.0	12.0	0.0	8.0	4.0	0.0
	9s24	43	ACC	25.9	9.3	23.3	0.0	4.7	7.0	0.0
			P171	26.2	20.9	4.7	2.3	2.3	9.3	0.0
			Double	—	2.3	16.3	2.3	4.7	14.0	0.0
	4A92cΔE	28	ACC	44.7	25.0	35.7	3.6	0.0	3.6	0.0
			P171	46.2	28.6	28.6	3.6	0.0	3.6	0.0
			Double	—	10.7	39.3	3.6	0.0	3.6	0.0
Xa5 & Xa23	92V1	38	Xa5	27.6	7.9	23.7	23.7	0.0	0.0	0.0
			Xa23	3.4	2.6	0.0	2.6	0.0	21.1	0.0
			Double	—	2.6	0.0	2.6	0.0	18.4	0.0
	4A92	45	Xa5	72.7	64.4	11.1	0.0	0.0	2.2	0.0
			Xa23	7.4	4.4	2.2	4.4	0.0	66.7	0.0
			Double	—	2.2	4.4	4.4	0.0	62.2	0.0
	492	42	Xa5	78.5	69.0	14.3	4.8	0.0	0.0	0.0
			Xa23	12.5	7.1	4.8	9.5	0.0	59.5	0.0
			Double	—	7.1	4.8	9.5	0.0	59.5	0.0
	924	30	Xa5	78.2	66.7	16.7	13.3	0.0	0.0	0.0
			Xa23	8.0	6.7	0.0	6.7	0.0	70.0	0.0
			Double	—	6.7	0.0	6.7	0.0	70.0	0.0
	9s2V1	—	Xa5	—	—	—	—	—	—	—
			Xa23	—	—	—	—	—	—	—
			Double	—	—	—	—	—	—	—
	9s42	44	Xa5	65.2	56.8	11.4	6.8	0.0	4.5	0.0
			Xa23	7.2	4.5	0.0	13.6	0.0	50.0	0.0
			Double	—	2.3	2.3	13.6	0.0	50.0	0.0
	9s24	40	Xa5	45.0	37.5	12.5	5.0	0.0	2.5	0.0
			Xa23	6.2	2.5	5.0	0.0	0.0	40.0	0.0
			Double	—	2.5	5.0	0.0	0.0	40.0	0.0

Target	PEs	No. of lines	Gene	Average percentage of desired reads (%)	Ratio of prime-edited lines (%)					
					Ho	He	Chi	Re	Indel	Sc
	4A92cΔE	29	Xa5	78.3	69.0	13.8	6.9	0.0	0.0	0.0
			Xa23	14.2	3.4	10.3	13.8	0.0	41.4	0.0
			Double	—	3.4	6.9	17.2	0.0	41.4	0.0
ROS & TT1	92V1	35	ROS	21.5	5.7	28.6	0.0	0.0	0.0	0.0
			TT1	0.0	0.0	0.0	0.0	5.7	0.0	0.0
			Double	—	0.0	0.0	0.0	5.7	0.0	0.0
	4A92	43	ROS	20.8	7.0	9.3	0.0	0.0	55.8	0.0
			TT1	0.0	0.0	0.0	0.0	0.0	2.3	0.0
			Double	—	0.0	0.0	0.0	0.0	2.3	0.0
	492	20	ROS	27.1	10.0	5.0	45.0	0.0	5.0	0.0
			TT1	3.5	0.0	5.0	5.0	20.0	0.0	0.0
			Double	—	0.0	0.0	10.0	15.0	5.0	0.0
	924	40	ROS	49.9	20.0	45.0	17.5	0.0	12.5	0.0
			TT1	3.9	2.5	2.5	0.0	17.5	0.0	0.0
			Double	—	0.0	0.0	5.0	17.5	0.0	0.0
	9s2V1	—	ROS	—	—	—	—	—	—	—
			TT1	—	—	—	—	—	—	—
			Double	—	—	—	—	—	—	—
	9s42	39	ROS	36.7	17.9	20.5	15.4	0.0	15.4	0.0
			TT1	0.0	0.0	0.0	0.0	2.6	0.0	0.0
			Double	—	0.0	0.0	0.0	2.6	0.0	0.0
	9s24	50	ROS	24.5	18.0	6.0	0.0	0.0	52.0	0.0
			TT1	2.5	2.0	0.0	0.0	24.0	0.0	0.0
			Double	—	0.0	0.0	0.0	4.0	22.0	0.0
	4A92cΔE	41	ROS	36.8	26.8	12.2	0.0	0.0	29.3	0.0
			TT1	5.3	2.4	4.9	2.4	4.9	2.4	0.0
			Double	—	0.0	0.0	0.0	0.0	17.1	0.0

The transgenic plants were analyzed by deep sequencing of PCR amplicons with 5% threshold. A line was deemed homozygous if 100% of deep-sequencing reads from that line represented a single type of mutation with the desired edits. Non-homozygous lines containing ≥ 45% desired edits were classified as heterozygous. Non-homozygous and non-heterozygous mutant lines were scored as Chimeric, Re, Sc, or Indel lines when the main mutation types were desired edits, Re byproducts (repair byproducts, where only a portion of the target bases had been edited in multi-base substitutions), Sc byproducts (scaffold-derived), or Indel byproducts, respectively. The mutation efficiency was calculated based on the ratio of the number of mutants to the total number of transgenic plants.

Methods S1

The sequences of Csy4-based prime editors, as well as the cloning cassettes for assembling two epegRNAs with two nicking sgRNAs, along with the final expression cassettes, are included in the Supplemental Sequences. All primers used in this study and sequences related to epegRNAs or nicking sgRNAs are also provided in Supplemental Sequences.

Vector construction

We separately cloned 9 synthetic fragments into a pUC57-derived vector, where the *Bsa*I site was disrupted, resulting in the creation of 9 pegRNA cloning vectors, including p2xPE3V1-P1Bb~P3Bb, p2xPE3RS-P1Bb~P3Bb and p2xPE3RSΔE-P1Bb~P3Bb.The *Hin*dIII-*Spe*I fragment of pG3H-2xPE3(Cao et al., 2024) was replaced with a synthetic fragment digested by *Hin*dIII and *Spe*I, generating pG3H-92-dV3.1. Subsequently, the *Xba*I-*Sbf*I fragment of pG3H-92-dV3.0 was replaced with a synthetic fragment digested by *Xba*I and *Sbf*I, yielding pG3H-4A92. The *Hin*dIII-*Spe*I fragment of pG3H-4A92 was replaced with a synthetic fragment, yielding pG3H-4A92-dV3RS. Additionally, we replaced the *Xba*I-*Sbf*I fragment of pG3H-4A92-dV3RS with a synthetic fragment, resulting in pG3H-492-dV3RS. Finally, the *Bsa*I-*Bsa*I fragment of pG3H-840KK2 (Jiang et al., 2022) was replaced with a synthetic fragment digested with *Bsa*I, resulting in pG3H-924, which was further modified by replacing its *Hin*dIII-*Spe*I fragment with that of pG3H-4A92-dV3RS, generating pG3H-924-dV3RS.

We replaced the *Bsa*I-*Bsa*I fragment of pG3H-840KK2 (Jiang et al., 2022) with a synthetic fragment digested with *Bsa*I, generating pG3H-KK840. Subsequently, the *Xba*I-*Sac*I fragment of pG3R23-PE3max-35C.5 (Jiang et al., 2022) was substituted with the *Xba*I-*Sac*I fragment of pG3H-KK840, leading to the creation of pG3R23-KK840. The *Hin*dIII-*Spe*I fragment of pG3R23-KK840 was replaced with a synthetic 2xPE3RS fragment digested with *Hin*dIII and *Spe*I, resulting in the formation of pG3R23-KK840-dV3RS. Additionally, the *Hin*dIII-*Spe*I fragment of pG3R23-KK840 was swapped with the *Hin*dIII-*Spe*I fragment of pG3H-92-dV3.1, producing pG3R23-KK840-dV3.1. The *Xba*I-*Sac*I fragment of pL2R4-OsMLH1dn was replaced with synthetic RT2, Csy4-RT2, or RT2-Csy4 fragments digested

with *Xba*I and *Sac*I, resulting in the creation of pL2R4-RT2, pL2R4-Csy4-RT2, and pL2R4-RT2-Csy4, respectively.

We used MultiSite Gateway technology to assemble pG3R23-KK840-dV3.1, pL2R4-RT2, and pL4L3-Hyg2, generating pG3H-9s2-dV3.1. Likewise, MultiSite Gateway technology was used to assemble pG3R23-KK840-dV3RS, pL4L3-Hyg2, and either pL2R4-RT2-Csy4 or pL2R4-Csy4-RT2, resulting in the creation of pG3H-9s24-dV3RS and pG3H-9s42-dV3RS.

To assemble 2xPE3V1-based PEs, including 92V1 and 9s2V1, we employed the *Bbs*I-based Golden Gate assembly method. This involved assembling p2xPE3V1-P1~P3 using p2xPE3V1-P1Bb~P3Bb cloning vectors and two short inserts generated by annealing two oligonucleotides. We then used the *Bsa*I-based Golden Gate method to assemble the 2xPE3V1-based PEs with p2xPE3V1-P1~P3 and one of the vectors pG3H-92-dV3.1 or pG3H-9s2-dV3.1.

For the assembly of 2xPE3RS-based PEs, including 4A92, 492, 924, 9s24 and 9s42, we first utilized the *Bbs*I-based Golden Gate method to construct p2xPE3RS-P1~P3 using the corresponding p2xPE3RS-P1Bb~P3Bb cloning vectors and two short inserts generated via oligo annealing. Subsequently, we applied the *Bsa*I-based Golden Gate assembly method to combine the p2xPE3V1-P1~P3 with one of the following vectors: pG3H-4A92-dV3RS, pG3H-492-dV3RS, pG3H-924-dV3RS, pG3H-9s24-dV3RS or pG3H-9s42-dV3RS.

To assemble 2xPE3RSΔE-based 4A92ΔE PEs, we first employed the *Bbs*I-based Golden Gate method to construct p2xPE3RSΔE-P1~P3 using the corresponding p2xPE3RSΔE-P1Bb~P3Bb cloning vectors, along with two short inserts generated by annealing oligonucleotides. Next, the *Bsa*I-based Golden Gate method was used to assemble the 4A92ΔE PEs by combining p2xPE3V1-P1~P3 with pG3H-4A92-dV3RS.

Rice transformation and prime editing analysis

We transformed duplex PE vectors into the engineered *Agrobacterium* strain LBA4404/pVS1-VIR2, generating strains that harbored the ternary vector system (Zhang et al., 2019). These strains were then used to transform callus cells derived from the rice cultivar Zhonghua11. To evaluate prime editing efficiency, we performed PCR amplification

of genomic DNA fragments spanning the target sites, using primers listed in Supplemental Sequences. The resulting PCR products were subjected to deep sequencing, with a sequencing depth of approximately 10,000 reads per amplicon. We utilized the Hi-TOM assay (Liu et al., 2019) with a 5% threshold to analyze the deep sequencing data.

We employed two methods to assess the prime-editing efficiency of each PE: a reads-based method and a sorting-based method. The reads-based approach enables precise quantification of fold change (Cao et al., 2024). In this method, the percentage of deep-sequencing reads with the desired edits was used to determine the editing efficiency for each PE in individual lines. The average percentage of such reads across all lines harboring the same PE was considered the editing efficiency for that PE.

For the sorting-based method, mutation efficiency was calculated by the ratio of mutant plants to the total number of transgenic plants harboring the same PE (Cao et al., 2024). A line was deemed homozygous if 100% of deep-sequencing reads from that line represented a single type of mutation with the desired edits. Non-homozygous lines containing ≥ 45% desired edits were classified as heterozygous. Non-homozygous and non-heterozygous mutant lines were scored as Chimeric, Re, Sc, or Indel lines when the main mutation types were desired edits, Re byproducts (repair byproducts, where only a portion of the target bases had been edited in multi-base substitutions), Sc byproducts (scaffold-derived), or Indel byproducts, respectively.

Supplemental Sequences

Supplemental Sequence 1. 4A92 (Csy4-P2A-Cas9n-RT2)

Csy4-P2A-bpNLS-SpCas9KK-H840A-2xSGGS-bpNLS-2xSGGS-RT2-SGGS-bpNLS-GSG-NLS$^{c\text{-}Myc}$

ATGGACCACTACCTGGACATCAGGCTCAGGCCGGACCCTGAGTTCCCGCCTGCCCAGCTGATGTCCGT
GCTGTTCGGCAAGCTGCATCAGGCTCTCGTTGCTCAGGGCGGCGATCGCATCGGCGTCTCATTCCCTGA
TCTGGACGAGTCTAGGAGCAGGCTGGGCGAGAGGCTCCGCATCCATGCCAGCGCTGACGACCTGAGGG
CTCTGCTCGCCAGGCCGTGGCTGGAGGGCCTCAGGGACCATCTCCAGTTCGGCGAGCCTGCTGTTGTG
CCACATCCAACACCGTACAGGCAGGTCAGCAGGGTGCAGGCCAAGTCCAATCCTGAGAGGCTGCGCAG
GCGCCTCATGAGGCGCCATGATCTGTCTGAGGAGGAGGCTAGGAAGCGCATCCCTGATACCGTTGCTA
GGGCGCTGGATCTGCCATTCGTCACACTGCGCTCACAGAGCACTGGCCAGCACTTCAGGCTGTTCATCA
GGCATGGCCCACTCCAGGTCACTGCTGAGGAGGGCGGCTTCACATGCTACGGCCTCTCCAAGGGCGGC
TTCGTTCCGTGGTTCGGCTCTGGCGCTACTAACTTCTCACTGCTGAAGCAGGCTGGCGATGTTGAGGAG
AATCCTGGCCCTATGAAGCGCACAGCCGATGGCAGCGAGTTCGAGTCACCTAAGAAGAAGCGCAAGGT

GGACAAGAAGTACTCGATCGGCCTCGATATTGGGACTAACTCTGTTGGCTGGGCCGTGATCACCGACGA
GTACAAGGTGCCCTCAAAGAAGTTCAAGGTCCTGGGCAACACCGATCGGCATTCCATCAAGAAGAATCT
CATTGGCGCTCTCCTGTTCGACAGCGGCGAGACGGCTGAGGCTACGCGGCTCAAGCGCACCGCCCGCA
GGCGGTACACGCGCAGGAAGAATCGCATCTGCTACCTGCAGGAGATTTTCTCCAACGAGATGGCGAAGG
TTGACGATTCTTTCTTCCACAGGCTGGAGGAGTCATTCCTCGTGGAGGAGGATAAGAAGCACGAGCGGC
ATCCAATCTTCGGCAACATTGTCGACGAGGTTGCCTACCACGAGAAGTACCCTACGATCTACCATCTGCG
GAAGAAGCTCGTGGACTCCACAGATAAGGCGGACCTCCGCCTGATCTACCTCGCTCTGGCCCACATGAT
TAAGTTCAGGGGCCATTTCCTGATCGAGGGGGATCTCAACCCGGACAATAGCGATGTTGACAAGCTGTT
CATCCAGCTCGTGCAGACGTACAACCAGCTCTTCGAGGAGAACCCCATTAATGCGTCAGGCGTCGACGC
GAAGGCTATCCTGTCCGCTAGGCTCTCGAAGTCTCGGAAGCTCGAGAACCTGATCGCCCAGCTGCCGG
GCGAGAAGAAGAACGGCCTGTTCGGGAATCTCATTGCGCTCAGCCTGGGGCTCACGCCCAACTTCAAGT
CGAATTTCGATCTCGCTGAGGACGCCAAGCTGCAGCTCTCCAAGGACACATACGACGATGACCTGGATA
ACCTCCTGGCCCAGATCGGCGATCAGTACGCGGACCTGTTCCTCGCTGCCAAGAATCTGTCGGACGCCA
TCCTCCTGTCTGATATTCTCAGGGTGAACACCGAGATTACGAAGGCTCCGCTCTCAGCCTCCATGATCAA
GCGCTACGACGAGCACCATCAGGATCTGACCCTCCTGAAGGCGCTGGTCAGGCAGCAGCTCCCCGAGA
AGTACAAGGAGATCTTCTTCGATCAGTCGAAGAACGGCTACGCTGGGTACATTGACGGCGGGGCCTCTC
AGGAGGAGTTCTACAAGTTCATCAAGCCGATTCTGGAGAAGATGGACGGCACGGAGGAGCTGCTGGTGA
AGCTCAAGCGCGAGGACCTCCTGAGGAAGCAGCGGACATTCGATAACGGCAGCATCCCACACCAGATTC
ATCTCGGGGAGCTGCACGCTATCCTGAGGAGGCAGGAGGACTTCTACCCTTTCCTCAAGGATAACCGCG
AGAAGATCGAGAAGATTCTGACTTTCAGGATCCCGTACTACGTCGGCCCACTCGCTAGGGGCAACTCCC
GCTTCGCTTGGATGACCCGCAAGTCAGAGGAGACGATCACGCCGTGGAACTTCGAGGAGGTGGTCGAC
AAGGGCGCTAGCGCTCAGTCGTTCATCGAGAGGATGACGAATTTCGACAAGAACCTGCCAAATGAGAAG
GTGCTCCCTAAGCACTCGCTCCTGTACGAGTACTTCACAGTCTACAACGAGCTGACTAAGGTGAAGTATG
TGACCGAGGGCATGAGGAAGCCGGCTTTCCTGTCTGGGGAGCAGAAGAAGGCCATCGTGGACCTCCTG
TTCAAGACCAACCGGAAGGTCACGGTTAAGCAGCTCAAGGAGGACTACTTCAAGAAGATTGAGTGCTTC
GATTCGGTCGAGATCTCTGGCGTTGAGGACCGCTTCAACGCCTCCCTGGGGACCTACCACGATCTCCTG
AAGATCATTAAGGATAAGGACTTCCTGGACAACGAGGAGAATGAGGATATCCTCGAGGACATTGTGCTGA
CACTCACTCTGTTCGAGGACCGGGAGATGATCGAGGAGCGCCTGAAGACTTACGCCCATCTCTTCGATG
ACAAGGTCATGAAGCAGCTCAAGAGGAGGAGGTACACCGGCTGGGGGAGGCTGAGCAGGAAGCTCATC
AACGGCATTCGGGACAAGCAGTCCGGGAAGACGATCCTCGACTTCCTGAAGAGCGATGGCTTCGCGAA
CCGCAATTTCATGCAGCTGATTCACGATGACAGCCTCACATTCAAGGAGGATATCCAGAAGGCTCAGGTG
AGCGGCCAGGGGGACTCGCTGCACGAGCATATCGCGAACCTCGCTGGCTCGCCAGCTATCAAGAAGGG
GATTCTGCAGACCGTGAAGGTTGTGGACGAGCTGGTGAAGGTCATGGGCAGGCACAAGCCTGAGAACA
TCGTCATTGAGATGGCCCGGGAGAATCAGACCACGCAGAAGGGCCAGAAGAACTCACGCGAGAGGATG
AAGAGGATCGAGGAGGGCATTAAGGAGCTGGGGTCCCAGATCCTCAAGGAGCACCCGGTGGAGAACAC
GCAGCTGCAGAATGAGAAGCTCTACCTGTACTACCTCCAGAATGGCCGCGATATGTATGTGGACCAGGA
GCTGGATATTAACAGGCTCAGCGATTACGACGTCGATGCCATCGTTCCACAGTCATTCCTGAAGGATGAC
TCCATTGACAACAAGGTCCTCACCAGGTCGGACAAGAACCGGGGCAAGTCTGATAATGTTCCTTCAGAG
GAGGTCGTTAAGAAGATGAAGAACTACTGGCGCCAGCTCCTGAATGCCAAGCTGATCACGCAGCGGAAG
TTCGATAACCTCACAAAGGCTGAGAGGGGCGGGCTCTCTGAGCTGGACAAGGCGGGCTTCATCAAGAG
GCAGCTGGTCGAGACACGGCAGATCACTAAGCACGTTGCGCAGATTCTCGACTCACGGATGAACACTAA
GTACGATGAGAATGACAAGCTGATCCGCGAGGTGAAGGTCATCACCCTGAAGTCAAAGCTCGTCTCCGA
CTTCAGGAAGGATTTCCAGTTCTACAAGGTTCGGGAGATCAACAATTACCACCATGCCCATGACGCGTAC
CTGAACGCGGTGGTCGGCACAGCTCTGATCAAGAAGTACCCAAAGCTCGAGAGCGAGTTCGTGTACGG

GGACTACAAGGTTTACGATGTGAGGAAGATGATCGCCAAGTCGGAGCAGGAGATTGGCAAGGCTACCGC
CAAGTACTTCTTCTACTCTAACATTATGAATTTCTTCAAGACAGAGATCACTCTGGCCAATGGCGAGATCC
GGAAGCGCCCCCTCATCGAGACGAACGGCGAGACGGGGGAGATCGTGTGGGACAAGGGCAGGGATTT
CGCGACCGTCAGGAAGGTTCTCTCCATGCCACAAGTGAATATCGTCAAGAAGACAGAGGTCCAGACTGG
CGGGTTCTCTAAGGAGTCAATTCTGCCTAAGCGGAACAGCGACAAGCTCATCGCCCGCAAGAAGGACTG
GGATCCGAAGAAGTACGGCGGGTTCGACAGCCCCACTGTGGCCTACTCGGTCCTGGTTGTGGCGAAGG
TTGAGAAGGGCAAGTCCAAGAAGCTCAAGAGCGTGAAGGAGCTGCTGGGGATCACGATTATGGAGCGC
TCCAGCTTCGAGAAGAACCCGATCGATTTCCTGGAGGCGAAGGGCTACAAGGAGGTGAAGAAGGACCT
GATCATTAAGCTCCCCAAGTACTCACTCTTCGAGCTGGAGAACGGCAGGAAGCGGATGCTGGCTTCCGC
TGGCGAGCTGCAGAAGGGGAACGAGCTGGCTCTGCCGTCCAAGTATGTGAACTTCCTCTACCTGGCCTC
CCACTACGAGAAGCTCAAGGGCAGCCCCGAGGACAACGAGCAGAAGCAGCTGTTCGTCGAGCAGCACA
AGCATTACCTCGACGAGATCATTGAGCAGATTTCCGAGTTCTCCAAGCGCGTGATCCTGGCCGACGCGA
ATCTGGATAAGGTCCTCTCCGCGTACAACAAGCACCGCGACAAGCCAATCAGGGAGCAGGCTGAGAATA
TCATTCATCTCTTCACCCTGACGAACCTCGGCGCCCCTGCTGCTTTCAAGTACTTCGACACAACTATCGA
TCGCAAGAGGTACACAAGCACTAAGGAGGTCCTGGACGCGACCCTCATCCACCAGTCGATTACCGGCCT
CTACGAGACGCGCATCGACCTGTCTCAGCTCGGGGGCGACTCAGGCGGCTCATCTGGCGGGTCAAAGC
GCACAGCCGACGGCTCTGAGTTCGAGAGCCCTAAGAAGAAGCGCAAGGTGTCAGGCGGCTCTTCAGGC
GGCAGCACCCTGAACATTGAGGACGAGTACCGGCTGCACGAGACGAGCAAGGAGCCAGACGTTTCGCT
CGGCAGCACTTGGCTCTCTGACTTCCCACAGGCTTGGGCCGAGACTGGCGGCATGGGCCTGGCCGTGC
GCCAGGCTCCACTGATCATCCCTCTGAAGGCGACCTCCACCCCGGTTTCTATTAAGCAGTACCCGATGA
GCCAGGAGGCCAGGCTGGGGATCAAGCCACACATTCAGCGGCTGCTGGACCAGGGCATCCTGGTGCCA
TGCCAGTCCCCGTGGAATACTCCGCTCCTGCCGGTGAAGAAGCCTGGGACAAACGACTACAGGCCGGT
TCAGGATCTCAGGGAGGTGAACAAGCGCGTGGAGGACATCCATCCGACAGTGCCGAACCCGTACAATCT
GCTGTCGGGCCTGCCTCCGAGCCACCAGTGGTACACCGTCCTGGACCTCAAGGACGCTTTCTTCTGCCT
GCGGCTGCACCCGACGTCTCAGCCGCTGTTCGCGTTCGAGTGGCGCGACCCAGAGATGGGCATTTCCG
GCCAGCTGACCTGGACACGCCTACCCCAGGGCTTCAAGAACTCCCCGACTCTCTTCAACGAGGCTCTCC
ACCGGGATCTCGCGGACTTCAGGATTCAGCATCCCGATCTGATCCTGCTCCAGTATGTTGACGACCTCCT
CCTGGCCGCGACGTCGGAGCTGGACTGCCAGCAGGGCACCCGGGCGCTGCTGCAGACACTGGGCAAT
CTGGGGTACCGCGCCTCTGCGAAGAAGGCGCAGATCTGCCAGAAGCAAGTGAAGTACCTGGGCTACCT
CCTGAAGGAGGGCCAGCGCTGGCTCACTGAGGCGAGGAAGGAGACTGTTATGGGCCAGCCCACTCCAA
AGACTCCGAGGCAGCTCAGGGAGTTCCTCGGCAAGGCTGGGTTCTGCCGCCTGTTCATCCCTGGGTTC
GCTGAGATGGCTGCGCCGCTCTACCCGCTGACTAAGCCGGGGACACTGTTCAACTGGGGGCCAGACCA
GCAGAAGGCGTACCAGGAGATTAAGCAGGCGCTGCTGACGGCCCCAGCGCTCGGCCTACCAGACCTGA
CGAAGCCGTTCGAGCTGTTCGTTGACGAGAAGCAGGGGTACGCGAAGGGCGTGCTGACACAGAAGCTG
GGGCCTTGGCGCCGCCCGGTCGCGTACCTGTCGAAGAAGCTGGACCCAGTCGCTGCTGGGTGGCCTC
CATGCCTCCGGATGGTCGCTGCTATTGCGGTTCTGACCAAGGATGCGGGGAAGCTCACAATGGGGCAG
CCTCTCGTGATCCTGGCTCCACATGCGGTGGAGGCGCTGGTGAAGCAGCCACCGGACCGGTGGCTGTC
GAACGCTCGGATGACACACTACCAGGCGCTCCTCCTCGATACAGACCGGGTTCAGTTCGGGCCTGTGGT
TGCTCTGAACCCAGCCACACTGCTGCCACTCCCTGAGGAGGGCCTCCAGCACAATTGCCTCGACATCCT
GGCTGAGGCGCACGGCACCCGCCCTGATCTCACCGACCAGCCTCTGCCAGATGCTGACCACACCTGGT
ACACGGATGGGTCCTCGCTGCTGCAGGAGGGCCAGAGGAAGGCGGGCGCCGCCGTCACCACAGAGAC
AGAGGTTATTTGGGCCAAGGCCCTACCGGCTGGCACCAGCGCCCAGCGCGCTGAGCTGATCGCGCTGA
CTCAGGCGCTGAAGATGGCCGAGGGGAAGAAGCTCAATGTTTACACCGACTCGCGGTACGCGTTCGCTA
CAGCTCACATTCATGGGGAGATCTACCGCCGGCGCGGGTGGCTGACTTCGGAGGGCAAGGAGATTAAG

AATAAGGACGAGATCCTGGCCCTGCTCAAGGCGCTGTTCCTGCCGAAGCGCCTCTCAATCATTCACTGC
CCGGGCCACCAGAAGGGCCATTCGGCCGAGGCTAGGGGCAATCGGATGGCTGACCAGGCGGCGCGGA
AGGCGGCTATCACCGAGACTCCCGATACATCTACCCTCCTGATCGAGAACTCGAGCCCATCTGGCGGCT
CTAAGCGGACTGCGGATGGGTCTGAGTTCGAGTCACCAAAGAAGAAGAGGAAGGTGGGCTCTGGCCCT
GCTGCTAAGCGCGTGAAGCTCGATTGA

RT2, PE2 RT.

Supplemental Sequence 2. 492 (Csy4-Cas9n-RT2)

Csy4-2xSGGS-bpNLS-2xSGGS-SpCas9KK-H840A-2xSGGS-bpNLS-2xSGGS-RT2-SGGS-bpNLS-GSG-NLS$^{c\text{-}Myc}$

ATGGACCACTACCTGGACATCAGGCTCAGGCCGGACCCTGAGTTCCCGCCTGCCCAGCTGATGTCCGT
GCTGTTCGGCAAGCTGCATCAGGCTCTCGTTGCTCAGGGCGGCGATCGCATCGGCGTCTCATTCCCTGA
TCTGGACGAGTCTAGGAGCAGGCTGGGCGAGAGGCTCCGCATCCATGCCAGCGCTGACGACCTGAGGG
CTCTGCTCGCCAGGCCGTGGCTGGAGGGCCTCAGGGACCATCTCCAGTTCGGCGAGCCTGCTGTTGTG
CCACATCCAACACCGTACAGGCAGGTCAGCAGGGTGCAGGCCAAGTCCAATCCTGAGAGGCTGCGCAG
GCGCCTCATGAGGCGCCATGATCTGTCTGAGGAGGAGGCTAGGAAGCGCATCCCTGATACCGTTGCTA
GGGCGCTGGATCTGCCATTCGTCACACTGCGCTCACAGAGCACTGGCCAGCACTTCAGGCTGTTCATCA
GGCATGGCCCACTCCAGGTCACTGCTGAGGAGGGCGGCTTCACATGCTACGGCCTCTCCAAGGGCGGC
TTCGTTCCGTGGTTCTCAGGCGGCTCATCTGGCGGGTCAAAGCGCACAGCCGACGGCTCTGAGTTCGA
GAGCCCTAAGAAGAAGCGCAAGGTGTCAGGCGGCTCTTCAGGCGGCTCAGACAAGAAGTACTCGATCG
GCCTCGATATTGGGACTAACTCTGTTGGCTGGGCCGTGATCACCGACGAGTACAAGGTGCCCTCAAAGA
AGTTCAAGGTCCTGGGCAACACCGATCGGCATTCCATCAAGAAGAATCTCATTGGCGCTCTCCTGTTCGA
CAGCGGCGAGAC//CAAGAGGTACACAAGCATAAGGAGGTCCTGGACGCGACCCTCATCCACCAGTCGAT
TACCGGCCTCTACGAGACGCGCATCGACCTGTCTCAGCTCGGGGGCGACTCAGGCGGCTCATCTGGCG
GGTCAAAGCGCACAGCCGACGGCTCTGAGTTCGAGAGCCCTAAGAAGAAGCGCAAGGTGTCAGGCGGC
TCTTCAGGCGGCAGCACCCTGAACATTGAGGACGAGTACCGGCTGCACGAGACGAGCAAGGAGCCAGA
CGTTTCGCTCGGCAGCACTTGGCTCTCTGACTTCCCACAG//AAGGGCCATTCGGCCGAGGCTAGGGGCA
ATCGGATGGCTGACCAGGCGGCGCGGAAGGCGGCTATCACCGAGACTCCCGATACATCTACCCTCCTG
ATCGAGAACTCGAGCCCATCTGGCGGCTCTAAGCGGACTGCGGATGGGTCTGAGTTCGAGTCACCAAAG
AAGAAGAGGAAGGTGGGCTCTGGCCCTGCTGCTAAGCGCGTGAAGCTCGATTGA

"//" indicates that the sequence is omitted for concision (see the full sequence in 4A92). RT2, PE2 RT.

Supplemental Sequence 3. 924 (Cas9n-RT2-Csy4)

bpNLS-SpCas9KK-H840A-2xSGGS-bpNLS-2xSGGS-RT2-XTEN-Csy4-SGGS-bpNLS-GSG-NLS$^{c\text{-}Myc}$

ATGAAGAGGACAGCCGATGGCAGCGAGTTCGAGAGCCCTAAGAAGAAGAGGAAGGTGGACAAGAAGTA
CTCGATCGGCCTCGATATTGGGACTAACTCTGTTGGCTGGGCCGTGATCACCGACGAGTACAAGGTGCC
CTCAAAGAAGTTCA//CGCAAGAGGTACACAAGCACTAAGGAGGTCCTGGACGCGACCCTCATCCACCAG
TCGATTACCGGCCTCTACGAGACGCGCATCGACCTGTCTCAGCTCGGGGGCGACTCAGGCGGCTCATC
TGGCGGGTCAAAGCGCACAGCCGACGGCTCTGAGTTCGAGAGCCCTAAGAAGAAGCGCAAGGTGTCAG
GCGGCTCTTCAGGCGGCAGCACCCTGAACATTGAGGACGAGTACCGGCTGCACGAGACGAGCAAGGAG
CCAGACGTTTCGCTCGGCAGCACTTGGCTCTCTGACTTCCCACAG//TTTGGGCCAAGGCCCTACCGGCT
GGCACCAGCGCCCAGCGCGCTGAGCTGATCGCGCTGACTCAGGCGCTGAAGATGGCTGAGGGCAAGA

AGCTCAACGTGTACACCGATAGCAGGTACGCCTTCGCTACAGCTCACATTCATGGCGAGATCTACCGCA
GACGCGGATGGCTGACTAGCGAGGGCAAGGAGATCAAGAACAAGGACGAGATCCTGGCTCTGCTCAAG
GCTCTGTTCCTGCCTAAGCGCCTGTCCATCATTCACTGCCCTGGCCACCAGAAGGGACATTCTGCCGAG
GCTCGCGGTAACCGGATGGCTGACCAGGCTGCTCGCAAGGCTGCTATCACCGAGACTCCCGATACATCT
ACACTGCTGATCGAGAACTCTAGCCCATCTGGTGGATCTAGCGGTGGATCATCTGGTAGCGAGACACCT
GGCACCAGCGAGTCTGCTACACCTGAGTCATCTGGTGGCAGCAGCGGTGGATCTATGGATCACTACCTG
GACATCAGGCTCAGGCCAGATCCTGAGTTCCCACCCGCTCAGCTGATGTCTGTGCTGTTCGGCAAGCTG
CATCAGGCTCTCGTTGCTCAGGGTGGCGATAGGATCGGTGTGTCATTCCCAGATCTCGATGAGTCTAGG
TCTAGGCTCGGTGAGAGGCTGCGCATCCATGCTAGCGCTGATGATCTCAGGGCTCTGCTCGCTAGGCCC
TGGCTCGAGGGACTCAGGGATCATCTGCAGTTCGGTGAGCCTGCTGTTGTGCCACATCCTACACCATAC
AGGCAGGTCAGCAGGGTGCAGGCCAAGTCCAATCCTGAGCGCCTGAGGCGCAGGCTCATGCGCAGGC
ATGATCTGTCTGAGGAGGAGGCTAGGAAGCGCATCCCTGATACCGTTGCTAGGGCTCTGGATCTGCCAT
TCGTCACACTGCGCTCACAGTCTACTGGCCAGCACTTCAGGCTGTTCATCAGGCATGGACCACTGCAGG
TCACTGCTGAGGAGGGTGGGTTCACATGCTACGGACTGAGCAAGGGTGGATTCGTGCCTTGGTTCAGCG
GTGGATCTAAGCGCACTGCTGATGGGTCTGAGTTCGAGTCACCCAAGAAGAAGCGCAAGGTCGGCTCTG
GCCCTGCTGCTAAGCGCGTGAAGCTCGATTGA

"//" indicates that the sequence is omitted for concision (see the full sequence in 4A92). RT2, PE2 RT.

Supplemental Sequence 4. 9s2 (Cas9n + RT2)

bpNLS-SpCas9KK-H840A-SGGS-bpNLS-GSG-NLS^{c-Myc}

ATGAAGAGGACAGCCGATGGCAGCGAGTTCGAGAGCCCTAAGAAGAAGAGGAAGGTGGACAAGAAGTA
CTCGATCGGCCTCGATATTGGGACTAACTCTGTTGGCTGGGCCGTGATCACCGACGAGTACAAGGTGCC
CTCAAAGAAGTTCAA//CGCAAGAGGTACACAAGCACTAAGGAGGTCCTGGACGCGACCCTCATCCACCA
GTCGATTACCGGCCTCTACGAGACGCGCATCGACCTGTCTCAGCTCGGGGGCGACTCTGGCGGCTCTA
AGCGGACTGCGGATGGGTCTGAGTTCGAGTCACCAAAGAAGAAGAGGAAGGTGGGCTCTGGCCCTGCT
GCTAAGCGCGTGAAGCTCGATTGA

bpNLS-2xSGGS-RT2-SGGS-bpNLS-GSG-NLS^{c-Myc}

AAGCGCACAGCCGACGGCTCTGAGTTCGAGAGCCCTAAGAAGAAGCGCAAGGTGTCAGGCGGCTCTTC
AGGCGGCAGCACCCTGAACATTGAGGACGAGTACCGGCTGCACGAGACGAGCAAGGAGCCAGACGTTT
CGCTCGGCAGCACTTGGCTCTCTGACTTCCCACAGG//CCAGAAGGGCCATTCGGCCGAGGCTAGGGGC
AATCGGATGGCTGACCAGGCGGCGCGGAAGGCGGCTATCACCGAGACTCCCGATACATCTACCCTCCT
GATCGAGAACTCGAGCCCATCTGGCGGCTCTAAGCGGACTGCGGATGGGTCTGAGTTCGAGTCACCAAA
GAAGAAGAGGAAGGTGGGCTCTGGCCCTGCTGCTAAGCGCGTGAAGCTCGATTGA

"//" indicates that the sequence is omitted for concision (see the full sequence in 4A92). RT2, PE2 RT.

Supplemental Sequence 5. 9s42 (Cas9n + Csy4-RT2)

bpNLS-SpCas9KK-H840A-SGGS-bpNLS-GSG-NLS^{c-Myc}

ATGAAGAGGACAGCCGATGGCAGCGAGTTCGAGAGCCCTAAGAAGAAGAGGAAGGTGGACAAGAAGTA
CTCGATCGGCCTCGATATTGGGACTAACTCTGTTGGCTGGGCCGTGATCACCGACGAGTACAAGGTGCC
CTCAAAGAAGTTCAA//CGCAAGAGGTACACAAGCACTAAGGAGGTCCTGGACGCGACCCTCATCCACCA
GTCGATTACCGGCCTCTACGAGACGCGCATCGACCTGTCTCAGCTCGGGGGCGACTCTGGCGGCTCTA
AGCGGACTGCGGATGGGTCTGAGTTCGAGTCACCAAAGAAGAAGAGGAAGGTGGGCTCTGGCCCTGCT

GCTAAGCGCGTGAAGCTCGATTGA

Csy4-2xSGGS-bpNLS-2xSGGS-RT2-SGGS-bpNLS-GSG-NLS$^{c\text{-}Myc}$
ATGGACCACTACCTGGACATCAGGCTCAGGCCGGACCCTGAGTTCCCGCCTGCCCAGCTGATGTCCGT
GCTGTTCGGCAAGCTGCATCAGGCTCTCGTTGCTCAGGGCGGCGATCGCATCGGCGTCTCATTCCCTGA
TCTGGACGAGTCTAGGAGCAGGCTGGGCGAGAGGCTCCGCATCCATGCCAGCGCTGACGACCTGAGGG
CTCTGCTCGCCAGGCCGTGGCTGGAGGGCCTCAGGGACCATCTCCAGTTCGGCGAGCCTGCTGTTGTG
CCACATCCAACACCGTACAGGCAGGTCAGCAGGGTGCAGGCCAAGTCCAATCCTGAGAGGCTGCGCAG
GCGCCTCATGAGGCGCCATGATCTGTCTGAGGAGGAGGCTAGGAAGCGCATCCCTGATACCGTTGCTA
GGGCGCTGGATCTGCCATTCGTCACACTGCGCTCACAGAGCACTGGCCAGCACTTCAGGCTGTTCATCA
GGCATGGCCCACTCCAGGTCACTGCTGAGGAGGGCGGCTTCACATGCTACGGCCTCTCCAAGGGCGGC
TTCGTTCCGTGGTTCTCAGGCGGCTCATCTGGCGGGTCAAAGCGCACAGCCGACGGCTCTGAGTTCGA
GAGCCCTAAGAAGAAGCGCAAGGTGTCAGGCGGCTCTTCAGGCGGCAGCACCCTGAACATTGAGGACG
AGTACCGGCTGCACGAGACGAGCAAGGAGCCAGACGTTTCGCTCGGCAGCACTTGGCTCTCTGACTTCC
CACAGG//CCAGAAGGGCCATTCGGCCGAGGCTAGGGGCAATCGGATGGCTGACCAGGCGGCGCGGAA
GGCGGCTATCACCGAGACTCCCGATACATCTACCCTCCTGATCGAGAACTCGAGCCCATCTGGCGGCTC
TAAGCGGACTGCGGATGGGTCTGAGTTCGAGTCACCAAAGAAGAAGAGGAAGGTGGGCTCTGGCCCTG
CTGCTAAGCGCGTGAAGCTCGAT

“//” indicates that the sequence is omitted for concision (see the full sequence in 4A92). RT2, PE2 RT.

Supplemental Sequence 6. 9s24 (Cas9n + RT2-Csy4)

bpNLS-SpCas9KK-H840A-SGGS-bpNLS-GSG-NLS$^{c\text{-}Myc}$
ATGAAGAGGACAGCCGATGGCAGCGAGTTCGAGAGCCCTAAGAAGAAGAGGAAGGTGGACAAGAAGTA
CTCGATCGGCCTCGATATTGGGACTAACTCTGTTGGCTGGGCCGTGATCACCGACGAGTACAAGGTGCC
CTCAAAGAAGTTCA//CGCAAGAGGTACACAAGCACTAAGGAGGTCCTGGACGCGACCCTCATCCACCAG
TCGATTACCGGCCTCTACGAGACGCGCATCGACCTGTCTCAGCTCGGGGGCGACTCTGGCGGCTCTAA
GCGGACTGCGGATGGGTCTGAGTTCGAGTCACCAAAGAAGAAGAGGAAGGTGGGCTCTGGCCCTGCTG
CTAAGCGCGTGAAGCTCGATTGA

bpNLS-RT2-XTEN-Csy4-SGGS-bpNLS-GSG-NLS$^{c\text{-}Myc}$
ATGAAGAGGACAGCCGATGGCAGCGAGTTCGAGAGCCCTAAGAAGAAGAGGAAGGTGACCCTGAACAT
TGAGGACGAGTACCGGCTGCACGAGACGAGCAAGGAGCCAGACGTTTCGCTCGGCAGCACTTGGCTCT
CTGACTTCCCACAG//TTTGGGCCAAGGCCCTACCGGCTGGCACCAGCGCCCAGCGCGCTGAGCTGATC
GCGCTGACTCAGGCGCTGAAGATGGCTGAGGGCAAGAAGCTCAACGTGTACACCGATAGCAGGTACGC
CTTCGCTACAGCTCACATTCATGGCGAGATCTACCGCAGACGCGGATGGCTGACTAGCGAGGGCAAGGA
GATCAAGAACAAGGACGAGATCCTGGCTCTGCTCAAGGCTCTGTTCCTGCCTAAGCGCCTGTCCATCATT
CACTGCCCTGGCCACCAGAAGGGACATTCTGCCGAGGCTCGCGGTAACCGGATGGCTGACCAGGCTGC
TCGCAAGGCTGCTATCACCGAGACTCCCGATACATCTACACTGCTGATCGAGAACTCTAGCCCATCTGGT
GGATCTAGCGGTGGATCATCTGGTAGCGAGACACCTGGCACCAGCGAGTCTGCTACACCTGAGTCATCT
GGTGGCAGCAGCGGTGGATCTATGGATCACTACCTGGACATCAGGCTCAGGCCAGATCCTGAGTTCCCA
CCCGCTCAGCTGATGTCTGTGCTGTTCGGCAAGCTGCATCAGGCTCTCGTTGCTCAGGGTGGCGATAGG
ATCGGTGTGTCATTCCCAGATCTCGATGAGTCTAGGTCTAGGCTCGGTGAGAGGCTGCGCATCCATGCT
AGCGCTGATGATCTCAGGGCTCTGCTCGCTAGGCCCTGGCTCGAGGGACTCAGGGATCATCTGCAGTTC
GGTGAGCCTGCTGTTGTGCCACATCCTACACCATACAGGCAGGTCAGCAGGGTGCAGGCCAAGTCCAAT

CCTGAGCGCCTGAGGCGCAGGCTCATGCGCAGGCATGATCTGTCTGAGGAGGAGGCTAGGAAGCGCAT
CCCTGATACCGTTGCTAGGGCTCTGGATCTGCCATTCGTCACACTGCGCTCACAGTCTACTGGCCAGCA
CTTCAGGCTGTTCATCAGGCATGGACCACTGCAGGTCACTGCTGAGGAGGGTGGGTTCACATGCTACGG
ACTGAGCAAGGGTGGATTCGTGCCTTGGTTCAGCGGTGGATCTAAGCGCACTGCTGATGGGTCTGAGTT
CGAGTCACCCAAGAAGAAGCGCAAGGTCGGCTCTGGCCCTGCTGCTAAGCGCGTGAAGCTCGATTGA
"//" indicates that the sequence is omitted for concision (see the full sequence in 4A92). RT2, PE2 RT.

Supplemental Sequence 7. Cloning cassette in p2xPE3V1-P1Bb

*Bsa*I-*Bbs*I-*Bbs*I-sgRNA-*Bbs*I-*Bbs*I-evopreQ1d2d0-HDV-tMet-*Bsa*I
TTCGCTACCTTAGGACCGTTATAGTTACGGTCTCA**GGTG**ATGTCTTCACAACCAATGAAGACAT**GTTT**AAGA
GCTATGCTGGAAACAGCATAGCAAGTTTAAATAAGGCTAGTCCGTTATCAACTTGAAAAAGTGGCACCGAG
TCG**GTGC**ATGTCTTCATGGTGGTGTGAAGACAA**CGGT**TCTATCTAGTTACGCGTTAAACCAACTAGAAAGG
CCGGCATGGTCCCAGCCTCCTCGCTGGCGCCGGCTGGGCAACATGCTTCGGCATGGCGAATGGGACAA
CAACAAATCAGAGTGGCGCAGCGGAAGCGTGGTGGGCCCATAACCCACAGGTCCCAGGATCGAAACCTG
GCTCT**GATA**TGAGACCAATAGGGATAACAGGGTAAT

Supplemental Sequence 8. Cloned sequence in p2xPE3V1-P1

*Bsa*I-Guide-sgRNA-rtT-PBS-Linker-evopreQ1-HDV-tMet-*Bsa*I
TTCGCTACCTTAGGACCGTTATAGTTACGGTCTCA**GGTG**CANNNNNNNNNNNNNNNNNNNN**GTTT**AAGAG
CTATGCTGGAAACAGCATAGCAAGTTTAAATAAGGCTAGTCCGTTATCAACTTGAAAAAGTGGCACCGAGT
CG**GTGC**NNNNNNNNNNNNNNNNNNNNNNNNNNNNNNNNNNNNNNNNNNNCG**CGGT**TCTATCTAGTTACGCGTTAA
ACCAACTAGAAAGGCCGGCATGGTCCCAGCCTCCTCGCTGGCGCCGGCTGGGCAACATGCTTCGGCATG
GCGAATGGGACAACAACAAATCAGAGTGGCGCAGCGGAAGCGTGGTGGGCCCATAACCCACAGGTCCC
AGGATCGAAACCTGGCTCT**GATA**TGAGACCAATAGGGATAACAGGGTAAT

Primers

oP1a-F	**GGTG**CANNNNNNNNNNNNNNNNNNNN	5'-Phosphorylation
oP1a-R	**AAAC**NNNNNNNNNNNNNNNNNNNNTG	5'-Phosphorylation
oP1b-F	**GTGC**NNNNNNNNNNNNNNNNNNNNNNNNNNNNNNNNNNNNNNNNNNCG	5'-Phosphorylation
oP1b-R	**ACCG**CGNNNNNNNNNNNNNNNNNNNNNNNNNNNNNNNNNNNNNNNNNNN	5'-Phosphorylation

Generation of short inserts by annealing two 5'-phosphorylated oligos (primers)
oP1a-F + oP1a-R = P1a
oP1b-F + oP1b-R = P1b
***Bbs*I-based Golden Gate Cloning**
p2xPE3V1-P1Bb + P1a + P1b = p2xPE3V1-P1

Supplemental Sequence 9. Cloning cassette in p2xPE3V1-P2Bb

*Bsa*I-*Bbs*I-*Bbs*I-sgRNA-*Bbs*I-*Bbs*I-evopreQ1d2d0-HDV-HSPt-OsU3p-*Bsa*I
TTCGCTACCTTAGGACCGTTATAGTTACGGTCTCA**GATA**ATGTCTTCACAACCAATGAAGACAT**GTTT**AAGA
GCTATGCTGGAAACAGCATAGCAAGTTTAAATAAGGCTAGTCCGTTATCAACTTGAAAAAGTGGCACCGAG
TCG**GTGC**ATGTCTTCATGGTGGTGTGAAGACAA**CGGT**TCTATCTAGTTACGCGTTAAACCAACTAGAAAGG
CCGGCATGGTCCCAGCCTCCTCGCTGGCGCCGGCTGGGCAACATGCTTCGGCATGGCGAATGGGACTTT
TTTTTGATATCTCCGGGGCTAATTGAATATGAAGATGAAGATGAAATATTTGGTGTGTCAAATAAAAAGCTGG

TGTGCTTAAGTTTGTGTTTTTTTCTTGGCTTGTTGTGTTATGAATTTGTGGCTTTTTCTAATATTAAATGAATG
TAAGATCTCATTATAATGAATAAACAAATGTTTCTATAATCCATTGTGAATGTTTTGTTGGATCTCTTCTGCAG
CATATAACTACTGTATGTGCTATGGTATGGACTATGGAATATGATTAAAGATAAAGTAATTCATCCAGGTCACC
AAGTTCTAGGATTTTCAGAACTGCAACTTATTTTATCAAGGAATCTTTAAACATACGAACAGATCACTTAAAG
TTCTTCTGAAGCAACTTAAAGTTATCAGGCTTGCATGGATCTTGGAGGAATCAGATGTGCAGTCAGGGACC
ATAGCACAAGACAGGCGTGTTCTACTGGTGCTACCAGCAAATGCTGGAAGCCGGGAACACTGGGTACGTT
GGAAACCACGTGATGTGAAGAAGTAAGATAAACTGTAGGAGAAAAGCATTTCGTAGTGGCCATGAAGCC
TTTCAGGACATGTATTGCAGTATGGGCCGGCCCATTACGCAATTGGACGACAACAAAGTCTAGTATTAGTAC
CACCTCGGCTATCCACATAGATCAAGCTGATTTAAAAGAGTTGTGCAGATGATCCG**TGGC**TGAGACCAATA
GGGATAACAGGGTAAT

Supplemental Sequence 10. Cloned sequence in p2xPE3V1-P2

*Bsa*I-Guide-sgRNA-rtT-PBS-linker-evopreQ1-HDV-HSPt-OsU3p-*Bsa*I

TTCGCTACCTTAGGACCGTTATAGTTACGGTCTCA**GATA**NNNNNNNNNNNNNNNNNNNN**GTTT**AAGAGCTA
TGCTGGAAACAGCATAGCAAGTTTAAATAAGGCTAGTCCGTTATCAACTTGAAAAAGTGGCACCGAGTCG**G**
TGCNNNNNNNNNNNNNNNNNNNNNNNNNNNNNNNNNNNNNNNNNNNNNCGCGGTTCTATCTAGTTACGCGTTAAACC
AACTAGAAAGGCCGGCATGGTCCCAGCCTCCTCGCTGGCGCCGGCTGGGCAACATGCTTCGGCATGGC
GAATGGGACTTTTTTTTGATATCTCCGGGGCTAATTGAATATGAAGATGAAGATGAAATATTTGGTGTGTCAA
ATAAAAAGCTGGTGTGCTTAAGTTTGTGTTTTTTTCTTGGCTTGTTGTGTTATGAATTTGTGGCTTTTTCTAA
TATTAAATGAATGTAAGATCTCATTATAATGAATAAACAAATGTTTCTATAATCCATTGTGAATGTTTTGTTGGA
TCTCTTCTGCAGCATATAACTACTGTATGTGCTATGGTATGGACTATGGAATATGATTAAAGATAAAGTAATTC
ATCCAGGTCACCAAGTTCTAGGATTTTCAGAACTGCAACTTATTTTATCAAGGAATCTTTAAACATACGAACA
GATCACTTAAAGTTCTTCTGAAGCAACTTAAAGTTATCAGGCTTGCATGGATCTTGGAGGAATCAGATGTGC
AGTCAGGGACCATAGCACAAGACAGGCGTGTTCTACTGGTGCTACCAGCAAATGCTGGAAGCCGGGAAC
ACTGGGTACGTTGGAAACCACGTGATGTGAAGAAGTAAGATAAACTGTAGGAGAAAAGCATTTCGTAGTGG
GCCATGAAGCCTTTCAGGACATGTATTGCAGTATGGGCCGGCCCATTACGCAATTGGACGACAACAAAGT
CTAGTATTAGTACCACCTCGGCTATCCACATAGATCAAAGCTGATTTAAAAGAGTTGTGCAGATGATCCG**TG**
GCTGAGACCAATAGGGATAACAGGGTAAT

Primers

oP2a-F	**GATA**NNNNNNNNNNNNNNNNNNNN	5'-Phosphorylation
oP2a-R	**AAAC**NNNNNNNNNNNNNNNNNNNN	5'-Phosphorylation
oP2b-F	**GTGC**NNNNNNNNNNNNNNNNNNNNNNNNNNNNNNNNNNNNNNNNNNNNNCG	5'-Phosphorylation
oP2b-R	**ACCG**CGNNNNNNNNNNNNNNNNNNNNNNNNNNNNNNNNNNNNNNNNNNNNN	5'-Phosphorylation

Generation of short inserts by annealing two 5'-phosphorylated oligos (primers)

oP2a-F + oP2a-R = P2a

oP2b-F + oP2b-R = P2b

BbsI-based Golden Gate Cloning

p2xPE3V1-P2Bb + P2a + P2b = p2xPE3V1-P2

Supplemental Sequence 11. Cloning cassette in p2xPE3V1-P3Bb

*Bsa*I-*Bbs*I-*Bbs*I-sgRNA-OsU3t-TaU3p-*Bbs*I-*Bbs*I-*Bsa*I

TTCGCTACCTTAGGACCGTTATAGTTACGGTCTCA**TGGC**ATGTCTTCACAACCAATGAAGACAT**GTTT**TAGA

GCTAGAAATAGCAAGTTAAAATAAGGCTAGTCCGTTATCAACTTGAAAAAGTGGCACCGAGTCGGTGCTTT
TTTTTTTCGTTTTGCATTGAGTTTTCTCCGTCGCATGTTTGCAGCATGAATCCAAACCACACGGAGTTCAAA
TTCCCACAGATTAAGGCTCGTCCGTCGCACAAGGTAATGTGTGAATATTATATCTGTCGTGCAAAATTGCCT
GGCCTGCACAATTGCTGTTATAGTTGGCGGCAGGGAGAGTTTTAACATTGACTAGCGTGCTGATAATTTGT
GAGAAATAATAATTGACAAGTAGATACTGACATTTGAGAAGAGCTTCTGAACTGTTATTAGTAACAAAAATGG
AAAGCTGATGCACGGAAAAAGGAAAGAAAAAGCCATACTTTTTTTTAGGTAGGAAAAGAAAAAGCCATACG
AGACTGATGTCTCTCAGATGGGCCGGGATCTGTCTATCTAGCAGGCAGCAGCCCACCAACCTCACGGGC
CAGCAATTACGAGTCCTTCTAAAAGCTCCCGCCGAGGGGCGCTGGCGCTGCTGTGCAGCAGCACGTCTA
ACATTAGTCCCACCTCGCCAGTTTACAGGGAGCAGAACCAGCTTATAAGCGGAGGCGCGGCACCAAG**AA**
GCATGTCTTCATGGTGGTGTGAAGACGG**TTTA**TGAGACCAATAGGGATAACAGGGTAAT

Supplemental Sequence 12. Cloned sequence in p2xPE3V1-P3

*Bsa*I-Guide-sgRNA-OsU3t-TaU3p-*Bsa*I

TTCGCTACCTTAGGACCGTTATAGTTACGGTCTCA**TGGC**<u>GNNNNNNNNNNNNNNNNNNN</u>**GTTT**TAGAGCT
AGAAATAGCAAGTTAAAATAAGGCTAGTCCGTTATCAACTTGAAAAAGTGGCACCGAGTCGGTGCTTTTTTT
TTTCGTTTTGCATTGAGTTTTCTCCGTCGCATGTTTGCAGCATGAATCCAAACCACACGGAGTTCAAATTCC
CACAGATTAAGGCTCGTCCGTCGCACAAGGTAATGTGTGAATATTATATCTGTCGTGCAAAATTGCCTGGC
CTGCACAATTGCTGTTATAGTTGGCGGCAGGGAGAGTTTTAACATTGACTAGCGTGCTGATAATTTGTGAG
AAATAATAATTGACAAGTAGATACTGACATTTGAGAAGAGCTTCTGAACTGTTATTAGTAACAAAAATGGAAA
GCTGATGCACGGAAAAAGGAAAGAAAAAGCCATACTTTTTTTTAGGTAGGAAAAGAAAAAGCCATACGAGA
CTGATGTCTCTCAGATGGGCCGGGATCTGTCTATCTAGCAGGCAGCAGCCCACCAACCTCACGGGCCAG
CAATTACGAGTCCTTCTAAAAGCTCCCGCCGAGGGGCGCTGGCGCTGCTGTGCAGCAGCACGTCTAACA
TTAGTCCCACCTCGCCAGTTTACAGGGAGCAGAACCAGCTTATAAGCGGAGGCGCGGCACCAAG**AAGC**<u>G</u>
<u>NNNNNNNNNNNNNNNNNNNGT</u>**TTTA**TGAGACCAATAGGGATAACAGGGTAAT

Primers

oP3a-F	**TGGC**GNNNNNNNNNNNNNNNNNNN	5'-Phosphorylation
oP3a-R	**AAAC**NNNNNNNNNNNNNNNNNNNC	5'-Phosphorylation
oP3b-F	**AAGC**GNNNNNNNNNNNNNNNNNNNGT	5'-Phosphorylation
oP3b-R	**TAAA**ACNNNNNNNNNNNNNNNNNNNC	5'-Phosphorylation

Generation of short inserts by annealing two 5'-phosphorylated oligos (primers)

oP3a-F + oP3a-R = P3a

oP3b-F + oP3b-R = P3b

BbsI-based Golden Gate Cloning

p2xPE3V1-P3Bb + P3a + P3b = p2xPE3V1-P3

Supplemental Sequence 13. Cloning cassette of 2xPE3V1

35S-CmYLCV-U6-tGly-*Bsa*I-*Bsa*I-sgRNAd2-TaU3t

ATGGAGTCAAAGATTCAAATAGAGGACCTAACAGAACTCGCCGTAAAGACTGGCGAACAGTTCATACAGAG
TCTCTTACGACTCAATGACAAGAAGAAAATCTTCGTCAACATGGTGGAGCACGACACACTTGTCTACTCCA
AAAATATCAAAGATACAGTCTCAGAAGACCAAAGGGCAATTGAGACTTTTCAACAAAGGGTAATATCCGGAA
ACCTCCTCGGATTCCATTGCCCAGCTATCTGTCACTTTATTGTGAAGATAGTGGAAAAGGAAGGTGGCTCC
TACAAATGCCATCATTGCGATAAAGGAAAGGCCATCGTTGAAGATGCCTCTGCCGACAGTGGTCCCAAAG

ATGGACCCCCACCCACGAGGAGCATCGTGGAAAAAGAAGACGTTCCAACCACGTCTTCAAAGCAAGTGG
ATTGATGTGATTGGCAGACATACTGTCCCACAAATGAAGATGGAATCTGTAAAAGAAAACGCGTGAAATAAT
GCGTCTGACAAAGGTTAGGTCGGCTGCCTTTAATCAATACCAAAGTGGTCCCTACCACGATGGAAAAACT
GTGCAGTCGGTTTGGCTTTTTCTGACGAACAAATAAGATTCGTGGCCGACAGGTGGGGGTCCACCATGTG
AAGGCATCTTCAGACTCCAATAATGGAGCAATGACGTAAGGGCTTACGAAATAAGTAAGGGTAGTTTGGGA
AATGTCCACTCACCCGTCAGTCTATAAATACTTAGCCCCTCCCTCATTGTTAAGGGAGCAAAATCTCAGAGA
GATAGTCCTAGAGAGAGAAAGAGAGCAAGTAGCCTAGAAGTAGTCAAGGCGGCGAAGTATTCAGGCACGT
GGCCAGGAAGAAGAAAAGCCAAGACGACGAAAACAGGTAAGAGCTAAGCATCTAGGTAAGTTGAAAACAA
TCTTCAAAAGTCCCACATCGCTTAGATAAGAAAACGAAGCTGAGTTTATATACAGCTAGAGTCGAAGTAGTG
ATTGAACAAA**GCAC**CAGTGGTCTAGTGGTAGAATAGTACCCTGCCACGGTACAGACCCGGGTTCGATTCC
CGGCT**GGTG**TGAGACCNNNNNNNNNNNNNNNNNNGGTCTCA**TTTA**GAGCTAGAAATAGCAAGTTAAAATA
AGGCTAGTCCGTTATCAACTTGAAAAAGTGGCACCGAGTCGGTGCTTTTTTTTTTGTCCTTCTGTTTTTTTA
GTCAGTCTCTTTTTTCAGAAGTACAACATCTT

Supplemental Sequence 14. Final cassette of 2xPE3V1

35S-CmYLCV-U6-tGly-Guide-sgRNA-rtT-PBS-linker-evopreQ1-HDV-tMet-Guide-sgRNA-rtT-PBS-linker-evopreQ1-HDV-polyT-HSPt-OsU3p-Guide-sgRNA-OsU3t-TaU3p-Guide-sgRNA-TaU3t

ATGGAGTCAAAGATTCAAATAGAGGACCTAACAGAACTCGCCGTAAAGACTGGCGAACAGTTCATACAGAG
TCTCTTACGACTCAATGACAAGAAGAAAATCTTCGTCAACATGGTGGAGCACGACACACTTGTCTACTCCA
AAAATATCAAAGATACAGTCTCAGAAGACCAAAGGGCAATTGAGACTTTTCAACAAAGGGTAATATCCGGAA
ACCTCCTCGGATTCCATTGCCCAGCTATCTGTCACTTTATTGTGAAGATAGTGGAAAAGGAAGGTGGCTCC
TACAAATGCCATCATTGCGATAAAGGAAAGGCCATCGTTGAAGATGCCTCTGCCGACAGTGGTCCCAAAG
ATGGACCCCCACCCACGAGGAGCATCGTGGAAAAAGAAGACGTTCCAACCACGTCTTCAAAGCAAGTGG
ATTGATGTGATTGGCAGACATACTGTCCCACAAATGAAGATGGAATCTGTAAAAGAAAACGCGTGAAATAAT
GCGTCTGACAAAGGTTAGGTCGGCTGCCTTTAATCAATACCAAAGTGGTCCCTACCACGATGGAAAAACT
GTGCAGTCGGTTTGGCTTTTTCTGACGAACAAATAAGATTCGTGGCCGACAGGTGGGGGTCCACCATGTG
AAGGCATCTTCAGACTCCAATAATGGAGCAATGACGTAAGGGCTTACGAAATAAGTAAGGGTAGTTTGGGA
AATGTCCACTCACCCGTCAGTCTATAAATACTTAGCCCCTCCCTCATTGTTAAGGGAGCAAAATCTCAGAGA
GATAGTCCTAGAGAGAGAAAGAGAGCAAGTAGCCTAGAAGTAGTCAAGGCGGCGAAGTATTCAGGCACGT
GGCCAGGAAGAAGAAAAGCCAAGACGACGAAAACAGGTAAGAGCTAAGCATCTAGGTAAGTTGAAAACAA
TCTTCAAAAGTCCCACATCGCTTAGATAAGAAAACGAAGCTGAGTTTATATACAGCTAGAGTCGAAGTAGTG
ATTGAACAAAGCACCAGTGGTCTAGTGGTAGAATAGTACCCTGCCACGGTACAGACCCGGGTTCGATTCC
CGGCT**GGTG**CANNNNNNNNNNNNNNNNNNNN**GTTT**AAGAGCTATGCTGGAAACAGCATAGCAAGTTTAAA
TAAGGCTAGTCCGTTATCAACTTGAAAAAGTGGCACCGAGTCG**GTGC**NNNNNNNNNNNNNNNNNNNNNN
NNNNNNNNNNNNNNNCG**CGGT**TCTATCTAGTTACGCGTTAAACCAACTAGAAAGGCCGGCATGGTCCCAG
CCTCCTCGCTGGCGCCGGCTGGGCAACATGCTTCGGCATGGCGAATGGGACAACAACAAATCAGAGTGG
CGCAGCGGAAGCGTGGTGGGCCCATAACCCACAGGTCCCAGGATCGAAACCTGGCTCT**GATA**NNNNNN
NNNNNNNNNNNNNN**GTTT**AAGAGCTATGCTGGAAACAGCATAGCAAGTTTAAATAAGGCTAGTCCGTTATC
AACTTGAAAAAGTGGCACCGAGTCG**GTGC**NNNNNNNNNNNNNNNNNNNNNNNNNNNNNNNNNNNNNCG
CGGTTCTATCTAGTTACGCGTTAAACCAACTAGAAAGGCCGGCATGGTCCCAGCCTCCTCGCTGGCGCCG
GCTGGGCAACATGCTTCGGCATGGCGAATGGGACTTTTTTTTGATATCTCCGGGGCTAATTGAATATGAAG
ATGAAGATGAAATATTTGGTGTGTCAAATAAAAAGCTGGTGTGCTTAAGTTTGTGTTTTTTTCTTGGCTTGTT
GTGTTATGAATTTGTGGCTTTTTCTAATATTAAATGAATGTAAGATCTCATTATAATGAATAAACAAATGTTTCT
ATAATCCATTGTGAATGTTTTGTTGGATCTCTTCTGCAGCATATAACTACTGTATGTGCTATGGTATGGACTAT

GGAATATGATTAAAGATAAAGTAATTCATCCAGGTCACCAAGTTCTAGGATTTTCAGAACTGCAACTTATTTT
ATCAAGGAATCTTTAAACATACGAACAGATCACTTAAAGTTCTTCTGAAGCAACTTAAAGTTATCAGGCTTG
CATGGATCTTGGAGGAATCAGATGTGCAGTCAGGGACCATAGCACAAG**ACAG**GCGTgTTCTACTGGTGCT
ACCAGCAAATGCTGGAAGCCGGGAACACTGGGTACGTTGGAAACCACGTGATGTGAAGAAGTAAGATAAA
CTGTAGGAGAAAAGCATTTCGTAGTGGGCCATGAAGCCTTTCAGGACATGTATTGCAGTATGGGCCGGCC
CATTACGCAATTGGACGACAACAAAGTCTAGTATTAGTACCACCTCGGCTATCCACATAGATCAAAGCTGAT
TTAAAAGAGTTGTGCAGATGATCCG**TGGC**GNNNNNNNNNNNNNNNNNNN**GTTT**TAGAGCTAGAAATAGCA
AGTTAAAATAAGGCTAGTCCGTTATCAACTTGAAAAAGTGGCACCGAGTCGGTGCTTTTTTTTTTCGTTTTG
CATTGAGTTTTCTCCGTCGCATGTTTGCAGCATGAATCCAAACCACACGGAGTTCAAATTCCCACAGATTAA
GGCTCGTCCGTCGCACAAGGTAATGTGTGAATATTATATCTGTCGTGCAAAATTGCCTGGCCTGCACAATT
GCTGTTATAGTTGGCGGCAGGGAGAGTTTTAACATTGACTAGCGTGCTGATAATTTGTGAGAAATAATAATT
GACAAGTAGATACTGACATTTGAGAAGAGCTTCTGAACTGTTATTAGTAACAAAAATGGAAAGCTGATGCAC
GGAAAAAGGAAAGAAAAAGCCATACTTTTTTTTAGGTAGGAAAAGAAAAAGCCATACGAGACTGATGTCTC
TCAGATGGGCCGGGATCTGTCTATCTAGCAGGCAGCAGCCCACCAACCTCACGGGCCAGCAATTACGAG
TCCTTCTAAAAGCTCCCGCCGAGGGGCGCTGGCGCTGCTGTGCAGCAGCACGTCTAACATTAGTCCCAC
CTCGCCAGTTTACAGGGAGCAGAACCAGCTTATAAGCGGAGGCGCGGCACCAAG**AAGC**GNNNNNNNNN
NNNNNNNNNN**GTTTA**GAGCTAGAAATAGCAAGTTAAAATAAGGCTAGTCCGTTATCAACTTGAAAAAGTG
GCACCGAGTCGGTGCTTTTTTTTTTGTCCTTCTGTTTTTTTAGTCAGTCTCTTTTTTCAGAAGTACAACATCT
T

BsaI-based Goden Gate cloning:

pG3H-92-dV3.1 + p2xPE3V1-P1 + p2xPE3V1-P2 + p2xPE3V1-P3 = p92-2xPE3V1 (92V1 for rice)

pG3H-9s2-dV3.1 + p2xPE3V1-P1 + p2xPE3V1-P2 + p2xPE3V1-P3 = p9s2-2xPE3V1 (9s2V1 for rice)

Supplemental Sequence 15. Cloning cassette in p2xPE3RS-P1Bb

*Bsa*I-*Bbs*I-*Bbs*I-sgRNA-*Bbs*I-*Bbs*I-evopreQ1d2d0-*Bsa*I

TTCGCTACCTTAGGACCGTTATAGTTACGGTCTCA**GCAG**ATGTCTTCACAACCAATGAAGACGC**GTTT**AAG
AGCTATGCTGGAAACAGCATAGCAAGTTTAAATAAGGCTAGTCCGTTATCAACTTGAAAAAGTGGCACCGA
GTCG**GTGC**ATGTCTTCATGGTGGTGTGAAGACAA**CGGT**TCTATCTAGTTACGCGTTAAACCAACTAGAAA**G**
TTCCGAGACCAATAGGGATAACAGGGTAATGTATGGT

Supplemental Sequence 16. Cloned sequence in p2xPE3RS-P1

*Bsa*I-Guide-sgRNA-rtT-PBS-linker-evopreQ1-*Bsa*I

TTCGCTACCTTAGGACCGTTATAGTTACGGTCTCA**GCAG**NNNNNNNNNNNNNNNNNNNN**GTTT**AAGAGCT
ATGCTGGAAACAGCATAGCAAGTTTAAATAAGGCTAGTCCGTTATCAACTTGAAAAAGTGGCACCGAGTCG
GTGCNNNNNNNNNNNNNNNNNNNNNNNNNNNNNNNNNNNNNCG**CGGT**TCTATCTAGTTACGCGTTAAAC
CAACTAGAAA**GTTC**CGAGACCAATAGGGATAACAGGGTAATGTATGGT

Primers

oP1a-F	**GCAG**NNNNNNNNNNNNNNNNNNNN	5'-Phosphorylation
oP1a-R	**AAAC**NNNNNNNNNNNNNNNNNNNN	5'-Phosphorylation
oP1b-F	**GTGC**NNNNNNNNNNNNNNNNNNNNNNNNNNNNNNNNNNNNNCG	5'-Phosphorylation
oP1b-R	**ACCG**CGNNNNNNNNNNNNNNNNNNNNNNNNNNNNNNNNNNNNN	5'-Phosphorylation

Generation of short inserts by annealing two 5'-phosphorylated oligos (primers)

oP1a-F + oP1a-R = P1a

oP1b-F + oP1b-R = P1b

BbsI-based Golden Gate Cloning

p2xPE3RS-P1Bb + P1a + P1b = p2xPE3RS-P1

Supplemental Sequence 17. Cloning cassette in p2xPE3RS-P2Bb

*Bsa*I-Csy4RS-*Bbs*I-*Bbs*I-sgRNA-*Bbs*I-*Bbs*I-*Bsa*I

TTCGCTACCTTAGGACCGTTATAGTTACGGTCTCA**GTTC**ACTGCCGTATAG**GCAG**ATGTCTTCACAACCAAT GAAGACGC**GTTT**AAGAGCTATGCTGGAAACAGCATAGCAAGTTTAAATAAGGCTAGTCCGTTATCAACTTG AAAAAGTGGCACCGAGTCG**GTGC**ATGTCTTCATGGTGGTGTGAAGACAA**CGGT**CGAGACCAATAGGGATA ACAGGGTAATGTATGGT

Supplemental Sequence 18. Cloned sequence in p2xPE3RS-P2

*Bsa*I-Csy4RS-Guide-sgRNA-rtT-PBS-linker-*Bsa*I

TTCGCTACCTTAGGACCGTTATAGTTACGGTCTCA**GTTC**ACTGCCGTATAG**GCAG**NNNNNNNNNNNNNNN NNNNN**GTTT**AAGAGCTATGCTGGAAACAGCATAGCAAGTTTAAATAAGGCTAGTCCGTTATCAACTTGAAAA AGTGGCACCGAGTCG**GTGC**NNNNNNNNNNNNNNNNNNNNNNNNNNNNNNNNNNNNNNNNNNNCG**CGGT**CGAGA CCAATAGGGATAACAGGGTAATGTATGGT

Primers

oP2a-F	**GCAG**NNNNNNNNNNNNNNNNNNNN	5'-Phosphorylation
oP2a-R	**AAAC**NNNNNNNNNNNNNNNNNNNN	5'-Phosphorylation
oP2b-F	**GTGC**NNNNNNNNNNNNNNNNNNNNNNNNNNNNNNNNNNNNNNNNNNNCG	5'-Phosphorylation
oP2b-R	**ACCG**CGNNNNNNNNNNNNNNNNNNNNNNNNNNNNNNNNNNNNNNNNNNN	5'-Phosphorylation

Generation of short inserts by annealing two 5'-phosphorylated oligos (primers)

oP2a-F + oP2a-R = P2a

oP2b-F + oP2b-R = P2b

BbsI-based Golden Gate Cloning

p2xPE3RS-P2Bb + P2a + P2b = p2xPE3RS-P2

Supplemental Sequence 19. Cloning cassette in p2xPE3RS-P3Bb

*Bsa*I-evopreQ1d2-Csy4RS- *Bbs*I-*Bbs*I -sgRNAd2d0-Csy4RSd2d0-*Bbs*I-*Bbs*I-*Bsa*I

TTCGCTACCTTAGGACCGTTATAGTTACGGTCTCA**CGGT**TCTATCTAGTTACGCGTTAAACCAACTAGAAAG TTCACTGCCGTATAG**GCAG**ATGTCTTCACAACCAATGAAGACGA**TTCA**GAGCTATGCTGGAAACAGCATAG CAAGTTGAAATAAGGCTAGTCCGTTATCAACTTGAAAAAGTGGCACCGAGTCGGTGCGTTCACTGCCGTAT **AGGC**ATGTCTTCATGGTGGTGTGAAGACGT**GTTT**CGAGACCAATAGGGATAACAGGGTAATGTATGGT

Supplemental Sequence 20. Cloned sequence in p2xPE3RS-P3

*Bsa*I-evopreQ1d2-Csy4RS-Guide-sgRNA-Csy4RS-Guide-*Bsa*I

TTCGCTACCTTAGGACCGTTATAGTTACGGTCTCA**CGGT**TCTATCTAGTTACGCGTTAAACCAACTAGAAAG TTCACTGCCGTATAG**GCAG**NNNNNNNNNNNNNNNNNNNNGT**TTCA**GAGCTATGCTGGAAACAGCATAGCA

AGTTGAAATAAGGCTAGTCCGTTATCAACTTGAAAAAGTGGCACCGAGTCGGTGCGTTCACTGCCGTATAG
GCAGNNNNNNNNNNNNNNNNNNNNGTTTCGAGACCAATAGGGATAACAGGGTAATGTATGGT

Primers

oP3a-F	**GCAG**NNNNNNNNNNNNNNNNNNNNGT	5'-Phosphorylation
oP3a-R	**TGAA**ACNNNNNNNNNNNNNNNNNNNN	5'-Phosphorylation
oP3b-F	**AGGC**AGNNNNNNNNNNNNNNNNNNNN	5'-Phosphorylation
oP3b-R	**AAAC**NNNNNNNNNNNNNNNNNNNNCT	5'-Phosphorylation

Generation of short inserts by annealing two 5'-phosphorylated oligos (primers)

oP3a-F + oP3a-R = P3a

oP3b-F + oP3b-R = P3b

BbsI-based Golden Gate Cloning

p2xPE3RS-P3Bb + P3a + P3b = p2xPE3RS-P3

Supplemental Sequence 21. Cloning cassette of 2xPE3RS

35S-CmYLCV-U6-Csy4RS-*Bsa*I-*Bsa*I-sgRNA-Csy4RS-polyT-HSPt

ATGGAGTCAAAGATTCAAATAGAGGACCTAACAGAACTCGCCGTAAAGACTGGCGAACAGTTCATACAGAG
TCTCTTACGACTCAATGACAAGAAGAAAATCTTCGTCAACATGGTGGAGCACGACACACTTGTCTACTCCA
AAAATATCAAAGATACAGTCTCAGAAGACCAAAGGGCAATTGAGACTTTTCAACAAAGGGTAATATCCGGAA
ACCTCCTCGGATTCCATTGCCCAGCTATCTGTCACTTTATTGTGAAGATAGTGGAAAAGGAAGGTGGCTCC
TACAAATGCCATCATTGCGATAAAGGAAAGGCCATCGTTGAAGATGCCTCTGCCGACAGTGGTCCCAAAG
ATGGACCCCCACCCACGAGGAGCATCGTGGAAAAAGAAGACGTTCCAACCACGTCTTCAAAGCAAGTGG
ATTGATGTGATTGGCAGACATACTGTCCCACAAATGAAGATGGAATCTGTAAAAGAAAACGCGTGAAATAAT
GCGTCTGACAAAGGTTAGGTCGGCTGCCTTTAATCAATACCAAAGTGGTCCCTACCACGATGGAAAAACT
GTGCAGTCGGTTTGGCTTTTTCTGACGAACAAATAAGATTCGTGGCCGACAGGTGGGGGTCCACCATGTG
AAGGCATCTTCAGACTCCAATAATGGAGCAATGACGTAAGGGCTTACGAAATAAGTAAGGGTAGTTTGGGA
AATGTCCACTCACCCGTCAGTCTATAAATACTTAGCCCCTCCCTCATTGTTAAGGGAGCAAAATCTCAGAGA
GATAGTCCTAGAGAGAGAAAGAGAGCAAGTAGCCTAGAAGTAGTCAAGGCGGCGAAGTATTCAGGCACGT
GGCCAGGAAGAAGAAAAGCCAAGACGACGAAAACAGGTAAGAGCTAAGCATCTAGGTAAGTTGAAAACAA
TCTTCAAAAGTCCCACATCGCTTAGATAAGAAAACGAAGCTGAGTTTATATACAGCTAGAGTCGAAGTAGTG
ATTGGTTCACTGCCGTATAG**GCAG**TGAGACCNNNNNNNNNNNNNNNNNNNGGTCTCA**GTTT**CAGAGCTATG
CTGGAAACAGCATAGCAAGTTGAAATAAGGCTAGTCCGTTATCAACTTGAAAAAGTGGCACCGAGTCGGT
GCGTTCACTGCCGTATAGGCAGTTTTTTTTGATATCTCCGGGGCTAATTGAATATGAAGATGAAGATGAAAT
ATTTGGTGTGTCAAATAAAAAGCTGGTGTGCTTAAGTTTGTGTTTTTTTCTTGGCTTGTTGTGTTATGAATTT
GTGGCTTTTTCTAATATTAAATGAATGTAAGATCTCATTATAATGAATAAACAAATGTTTCTATAATCCATTGTG
AATGTTTTGTTGGATCTCTTCTGCAGCATATAACTACTGTATGTGCTATGGTATGGACTATGGAATATGATTAA
AGATAA

Supplemental Sequence 22. Final cassette of 2xPE3RS

35S-CmYLCV-U6-Csy4RS-Guide-sgRNA-rtT-PBS-linker-evopreQ1-Csy4RS-Guide-sgRNA-rtT-PBS-linker-evopreQ1-Csy4RS-Guide-sgRNA-Csy4RS-Guide-sgRNA-Csy4RS-polyT-HSPt

ATGGAGTCAAAGATTCAAATAGAGGACCTAACAGAACTCGCCGTAAAGACTGGCGAACAGTTCATACAGAG
TCTCTTACGACTCAATGACAAGAAGAAAATCTTCGTCAACATGGTGGAGCACGACACACTTGTCTACTCCA

AAAATATCAAAGATACAGTCTCAGAAGACCAAAGGGCAATTGAGACTTTTCAACAAAGGGTAATATCCGGAA
ACCTCCTCGGATTCCATTGCCCAGCTATCTGTCACTTTATTGTGAAGATAGTGGAAAAGGAAGGTGGCTCC
TACAAATGCCATCATTGCGATAAAGGAAAGGCCATCGTTGAAGATGCCTCTGCCGACAGTGGTCCCAAAG
ATGGACCCCCACCCACGAGGAGCATCGTGGAAAAAGAAGACGTTCCAACCACGTCTTCAAAGCAAGTGG
ATTGATGTGATTGGCAGACATACTGTCCCACAAATGAAGATGGAATCTGTAAAAGAAAACGCGTGAAATAAT
GCGTCTGACAAAGGTTAGGTCGGCTGCCTTTAATCAATACCAAAGTGGTCCCTACCACGATGGAAAAACT
GTGCAGTCGGTTTGGCTTTTTCTGACGAACAAATAAGATTCGTGGCCGACAGGTGGGGGTCCACCATGTG
AAGGCATCTTCAGACTCCAATAATGGAGCAATGACGTAAGGGCTTACGAAATAAGTAAGGGTAGTTTGGGA
AATGTCCACTCACCCGTCAGTCTATAAATACTTAGCCCCTCCCTCATTGTTAAGGGAGCAAAATCTCAGAGA
GATAGTCCTAGAGAGAGAAAGAGAGCAAGTAGCCTAGAAGTAGTCAAGGCGGCGAAGTATTCAGGCACGT
GGCCAGGAAGAAGAAAAGCCAAGACGACGAAAACAGGTAAGAGCTAAGCATCTAGGTAAGTTGAAAACAA
TCTTCAAAAGTCCCACATCGCTTAGATAAGAAAACGAAGCTGAGTTTATATACAGCTAGAGTCGAAGTAGTG
ATTGGTTCACTGCCGTATAG**GCAG**NNNNNNNNNNNNNNNNNNNN**GTTT**AAGAGCTATGCTGGAAACAGCA
TAGCAAGTTTAAATAAGGCTAGTCCGTTATCAACTTGAAAAAGTGGCACCGAGTCG**GTGC**NNNNNNNNNN
NNNNNNNNNNNNNNNNNNNNNNNNNNNCG**CGGT**TCTATCTAGTTACGCGTTAAACCAACTAGAAAG**TTCA**
CTGCCGTATA**GGCA**GNNNNNNNNNNNNNNNNNNNN**GTTT**AAGAGCTATGCTGGAAACAGCATAGCAAGTT
TAAATAAGGCTAGTCCGTTATCAACTTGAAAAAGTGGCACCGAGTCG**GTGC**NNNNNNNNNNNNNNNNNNN
NNNNNNNNNNNNNNNNNNCG**CGGT**TCTATCTAGTTACGCGTTAAACCAACTAGAAAG**TTCA**CTGCCGTAT
AGGCAGNNNNNNNNNNNNNNNNNNNNGTTTCAGAGCTATGCTGGAAACAGCATAGCAAGTTGAAATAAG
GCTAGTCCGTTATCAACTTGAAAAAGTGGCACCGAGTCGGTGCGTTCACTGCCGTA**TAGG**CAGNNNNNNN
NNNNNNNNNNNNN**GTTT**CAGAGCTATGCTGGAAACAGCATAGCAAGTTGAAATAAGGCTAGTCCGTTATCA
ACTTGAAAAAGTGGCACCGAGTCGGTGCGTTCACTGCCGTATAGGCAGTTTTTTTTGATATCTCCGGGGCT
AATTGAATATGAAGATGAAGATGAAATATTTGGTGTGTCAAATAAAAAGCTGGTGTGCTTAAGTTTGTGTTTT
TTTCTTGGCTTGTTGTGTTATGAATTTGTGGCTTTTTCTAATATTAAATGAATGTAAGATCTCATTATAATGAAT
AAACAAATGTTTCTATAATCCATTGTGAATGTTTTGTTGGATCTCTTCTGCAGCATATAACTACTGTATGTGCT
ATGGTATGGACTATGGAATATGATTAAAGATAA

BsaI-based Goden Gate cloning:

pG3H-4A92-dV3RS + p2xPE3RS-P1 + p2xPE3RS-P2 + p2xPE3RS-P3 = 4A92-2xPE3RS (4A92 for rice)
pG3H-492-dV3RS + p2xPE3RS-P1 + p2xPE3RS-P2 + p2xPE3RS-P3 = 492-2xPE3RS (492 for rice)
pG3H-924-dV3RS + p2xPE3RS-P1 + p2xPE3RS-P2 + p2xPE3RS-P3 = 924-2xPE3RS (924 for rice)
pG3H-9s24-dV3RS + p2xPE3RS-P1 + p2xPE3RS-P2 + p2xPE3RS-P3 = 9s24-2xPE3RS (9s24 for rice)
pG3H-9s42-dV3RS + p2xPE3RS-P1 + p2xPE3RS-P2 + p2xPE3RS-P3 = 9s42-2xPE3RS (9s42 for rice)
pG3H-4A96c-dV3RS + p2xPE3RS-P1 + p2xPE3RS-P2 + p2xPE3RS-P3 = 4A96c-2xPE3RS (4A96c for rice)

Supplemental Sequence 23. Cloning cassette in p2xPE3RS∆E-P1Bb

*Bsa*I-*Bbs*I-*Bbs*I-sgRNA-*Bbs*I-*Bbs*I-Csy4RSd1d1-*Bsa*I

TTCGCTACCTTAGGACCGTTATAGTTACGGTCTCA**GCAG**TTGTCTTCACAACCAATGAAGACAT**GTTT**AAGA
GCTATGCTGGAAACAGCATAGCAAGTTTAAATAAGGCTAGTCCGTTATCAACTTGAAAAAGTGGCACCGAG
TCG**GTGC**ATGTCTTCATGGTGGTGTGAAGACAA**TTCA**CTGCCGTATA**GGCA**CGAGACCAATAGGGATAAC
AGGGTAAT

Supplemental Sequence 24. Cloned sequence in p2xPE3RS∆E-P1

*Bsa*I-Guide-sgRNA-rtT-PBS-Linker-Csy4d0d1-*Bsa*I

TTCGCTACCTTAGGACCGTTATAGTTACGGTCTCA**GCAG**NNNNNNNNNNNNNNNNNNNN**GTTT**AAGAGCT
ATGCTGGAAACAGCATAGCAAGTTTAAATAAGGCTAGTCCGTTATCAACTTGAAAAAGTGGCACCGAGTCG
GTGCNNNNNNNNNNNNNNNNNNNNNNNNNNNNNNNNNNNNNG**TTCA**CTGCCGTATA**GGCA**CGAGACCA
ATAGGGATAACAGGGTAAT

Primers

oP1a-F	**GCAG**NNNNNNNNNNNNNNNNNNNN	5'-Phosphorylation
oP1a-R	**AAAC**NNNNNNNNNNNNNNNNNNNN	5'-Phosphorylation
oP1b-F	**GTGC**NNNNNNNNNNNNNNNNNNNNNNNNNNNNNNNNNNNNNG	5'-Phosphorylation
oP1b-R	**TGAA**CNNNNNNNNNNNNNNNNNNNNNNNNNNNNNNNNNNNNN	5'-Phosphorylation

Generation of short inserts by annealing two 5'-phosphorylated oligos (primers)

oP1a-F + oP1a-R = P1a

oP1b-F + oP1b-R = P1b

BbsI-based Golden Gate Cloning

p2xPE3RSΔE-P1Bb + P1a + P1b = p2xPE3RSΔE-P1

Supplemental Sequence 25. Cloning cassette in p2xPE3RSΔE-P2Bb

*Bsa*I-*Bbs*I-*Bbs*I-sgRNA-*Bbs*I-*Bbs*I-Csy4RSd1d2-*Bsa*I

TTCGCTACCTTAGGACCGTTATAGTTACGGTCTCA**GGCA**TTGTCTTCACAACCAATGAAGACAT**GTTT**AAGA
GCTATGCTGGAAACAGCATAGCAAGTTTAAATAAGGCTAGTCCGTTATCAACTTGAAAAAGTGGCACCGAG
TCG**GTGC**ATGTCTTCATGGTGGTGTGAAGACAA**TTCA**CTGCCGTAT**AGGC**TGAGACCAATAGGGATAACA
GGGTAAT

Supplemental Sequence 26. Cloned sequence in p2xPE3RSΔE-P2

*Bsa*I-Guide-sgRNA-rtT-PBS-Linker-Csy4d0d2-*Bsa*I

TTCGCTACCTTAGGACCGTTATAGTTACGGTCTCA**GGCA**GNNNNNNNNNNNNNNNNNNNN**GTTT**AAGAGC
TATGCTGGAAACAGCATAGCAAGTTTAAATAAGGCTAGTCCGTTATCAACTTGAAAAAGTGGCACCGAGTC
G**GTGC**NNNNNNNNNNNNNNNNNNNNNNNNNNNNNNNNNNNNNG**TTCA**CTGCCGTAT**AGGC**TGAGACCA
ATAGGGATAACAGGGTAAT

Primers

oP2a-F	**GGCA**GNNNNNNNNNNNNNNNNNNNN	5'-Phosphorylation
oP2a-R	**AAAC**NNNNNNNNNNNNNNNNNNNNC	5'-Phosphorylation
oP2b-F	**GTGC**NNNNNNNNNNNNNNNNNNNNNNNNNNNNNNNNNNNNNG	5'-Phosphorylation
oP2b-R	**TGAA**CNNNNNNNNNNNNNNNNNNNNNNNNNNNNNNNNNNNNN	5'-Phosphorylation

Generation of short inserts by annealing two 5'-phosphorylated oligos (primers)

oP2a-F + oP2a-R = P2a

oP2b-F + oP2b-R = P2b

BbsI-based Golden Gate Cloning

p2xPE3RSΔE-P2Bb + P2a + P2b = p2xPE3RSΔE-P2

Supplemental Sequence 27. Cloning cassette in p2xPE3RSΔE-P3Bb

*Bsa*I-*Bbs*I-*Bbs*I-sgRNA-Csy4RSd0d3-*Bbs*I-*Bbs*I-*Bsa*I

TTCGCTACCTTAGGACCGTTATAGTTACGGTCTCA**AGGC**ATGTCTTCACAACCAATGAAGACGA**TTCA**GAG
CTATGCTGGAAACAGCATAGCAAGTTGAAATAAGGCTAGTCCGTTATCAACTTGAAAAAGTGGCACCGAGT
CGGTGCGTTCACTGCCGTA**TAGG**ATGTCTTCATGGTGGTGTGAAGACGT**GTTT**CGAGACCAATAGGGATA
ACAGGGTAAT

Supplemental Sequence 28. Cloned sequence in p2xPE3RSΔE-P3

*Bsa*I-Guide-sgRNA-Csy4RS-Guide-*Bsa*I

TTCGCTACCTTAGGACCGTTATAGTTACGGTCTCA**AGGC**AGNNNNNNNNNNNNNNNNNNNNG**TTTCA**GAG
CTATGCTGGAAACAGCATAGCAAGTTGAAATAAGGCTAGTCCGTTATCAACTTGAAAAAGTGGCACCGAGT
CGGTGCGTTCACTGCCGTA**TAGG**CAGNNNNNNNNNNNNNNNNNNNN**GTTT**CGAGACCAATAGGGATAAC
AGGGTAAT

Primers

oP3a-F	**AGGC**AGNNNNNNNNNNNNNNNNNNNNGT	5'-Phosphorylation
oP3a-R	**TGAA**ACNNNNNNNNNNNNNNNNNNNNCT	5'-Phosphorylation
oP3b-F	**TAGG**CAGNNNNNNNNNNNNNNNNNNNN	5'-Phosphorylation
oP3b-R	**AAAC**NNNNNNNNNNNNNNNNNNNNCTG	5'-Phosphorylation

Generation of short inserts by annealing two 5'-phosphorylated oligos (primers)

oP3a-F + oP3a-R = P3a

oP3b-F + oP3b-R = P3b

BbsI-based Golden Gate Cloning

p2xPE3RSΔE-P3Bb + P3a + P3b = p2xPE3RSΔE-P3

Supplemental Sequence 29. Cloning cassette of 2xPE3RSΔE

35S-CmYLCV-U6- Csy4RS-*Bsa*I-*Bsa*I- Csy4RS-sgRNA-polyT-HSPt

ATGGAGTCAAAGATTCAAATAGAGGACCTAACAGAACTCGCCGTAAAGACTGGCGAACAGTTCATACAGAG
TCTCTTACGACTCAATGACAAGAAGAAAATCTTCGTCAACATGGTGGAGCACGACACACTTGTCTACTCCA
AAAATATCAAAGATACAGTCTCAGAAGACCAAAGGGCAATTGAGACTTTTCAACAAAGGGTAATATCCGGAA
ACCTCCTCGGATTCCATTGCCCAGCTATCTGTCACTTTATTGTGAAGATAGTGGAAAAGGAAGGTGGCTCC
TACAAATGCCATCATTGCGATAAAGGAAAGGCCATCGTTGAAGATGCCTCTGCCGACAGTGGTCCCAAAG
ATGGACCCCCACCCACGAGGAGCATCGTGGAAAAAGAAGACGTTCCAACCACGTCTTCAAAGCAAGTGG
ATTGATGTGATTGGCAGACATACTGTCCCACAAATGAAGATGGAATCTGTAAAAGAAAACGCGTGAAATAAT
GCGTCTGACAAAGGTTAGGTCGGCTGCCTTTAATCAATACCAAAGTGGTCCCTACCACGATGGAAAAACT
GTGCAGTCGGTTTGGCTTTTTCTGACGAACAAATAAGATTCGTGGCCGACAGGTGGGGGTCCACCATGTG
AAGGCATCTTCAGACTCCAATAATGGAGCAATGACGTAAGGGCTTACGAAATAAGTAAGGGTAGTTTGGGA
AATGTCCACTCACCCGTCAGTCTATAAATACTTAGCCCCTCCCTCATTGTTAAGGGAGCAAAATCTCAGAGA
GATAGTCCTAGAGAGAGAAAGAGAGCAAGTAGCCTAGAAGTAGTCAAGGCGGCGAAGTATTCAGGCACGT
GGCCAGGAAGAAGAAAAGCCAAGACGACGAAAACAGGTAAGAGCTAAGCATCTAGGTAAGTTGAAAACAA
TCTTCAAAAGTCCCACATCGCTTAGATAAGAAAACGAAGCTGAGTTTATATACAGCTAGAGTCGAAGTAGTG
ATTGGTTCACTGCCGTATAG**GCAG**TGAGACCNNNNNNNNNNNNNNNNNNNGGTCTCA**GTTT**CAGAGCTATG
CTGGAAACAGCATAGCAAGTTGAAATAAGGCTAGTCCGTTATCAACTTGAAAAAGTGGCACCGAGTCGGT
GCGTTCACTGCCGTATAGGCAGTTTTTTTTGATATCTCCGGGGCTAATTGAATATGAAGATGAAGATGAAAT

ATTTGGTGTGTCAAATAAAAAGCTGGTGTGCTTAAGTTTGTGTTTTTTTCTTGGCTTGTTGTGTTATGAATTT
GTGGCTTTTTCTAATATTAAATGAATGTAAGATCTCATTATAATGAATAAACAAATGTTTCTATAATCCATTGTG
AATGTTTTGTTGGATCTCTTCTGCAGCATATAACTACTGTATGTGCTATGGTATGGACTATGGAATATGATTAA
AGATAA

Supplemental Sequence 30. Final cassette of 2xPE3RSΔE

35S-CmYLCV-U6-Csy4RS-Guide-sgRNA-rtT-PBS-linker-Csy4RS-Guide-sgRNA-rtT-PBS-linker-Csy4RS-Guide-sgRNA- Csy4RS-Guide-sgRNA-Csy4RS-polyT-HSPt

ATGGAGTCAAAGATTCAAATAGAGGACCTAACAGAACTCGCCGTAAAGACTGGCGAACAGTTCATACAGAG
TCTCTTACGACTCAATGACAAGAAGAAAATCTTCGTCAACATGGTGGAGCACGACACACTTGTCTACTCCA
AAAATATCAAAGATACAGTCTCAGAAGACCAAAGGGCAATTGAGACTTTTCAACAAAGGGTAATATCCGGAA
ACCTCCTCGGATTCCATTGCCCAGCTATCTGTCACTTTATTGTGAAGATAGTGGAAAAGGAAGGTGGCTCC
TACAAATGCCATCATTGCGATAAAGGAAAGGCCATCGTTGAAGATGCCTCTGCCGACAGTGGTCCCAAAG
ATGGACCCCCACCCACGAGGAGCATCGTGGAAAAAGAAGACGTTCCAACCACGTCTTCAAAGCAAGTGG
ATTGATGTGATTGGCAGACATACTGTCCCACAAATGAAGATGGAATCTGTAAAAGAAAACGCGTGAAATAAT
GCGTCTGACAAAGGTTAGGTCGGCTGCCTTTAATCAATACCAAAGTGGTCCCTACCACGATGGAAAAACT
GTGCAGTCGGTTTGGCTTTTTCTGACGAACAAATAAGATTCGTGGCCGACAGGTGGGGGTCCACCATGTG
AAGGCATCTTCAGACTCCAATAATGGAGCAATGACGTAAGGGCTTACGAAATAAGTAAGGGTAGTTTGGGA
AATGTCCACTCACCCGTCAGTCTATAAATACTTAGCCCCTCCCTCATTGTTAAGGGAGCAAAATCTCAGAGA
GATAGTCCTAGAGAGAGAAAGAGAGCAAGTAGCCTAGAAGTAGTCAAGGCGGCGAAGTATTCAGGCACGT
GGCCAGGAAGAAGAAAAGCCAAGACGACGAAAACAGGTAAGAGCTAAGCATCTAGGTAAGTTGAAAACAA
TCTTCAAAAGTCCCACATCGCTTAGATAAGAAAACGAAGCTGAGTTTATATACAGCTAGAGTCGAAGTAGTG
ATTGGTTCACTGCCGTATAGGCAGNNNNNNNNNNNNNNNNNNNN**GTTT**AAGAGCTATGCTGGAAACAGCA
TAGCAAGTTTAAATAAGGCTAGTCCGTTATCAACTTGAAAAAGTGGCACCGAGTCG**GTGC**NNNNNNNNNN
NNNNNNNNNNNNNNNNNNNNNNNNNNNNNNNGTTCACTGCCGTATAGGCAGNNNNNNNNNNNNNNNNNNNN**G**
TTTAAGAGCTATGCTGGAAACAGCATAGCAAGTTTAAATAAGGCTAGTCCGTTATCAACTTGAAAAAGTGGC
ACCGAGTCG**GTGC**NNNNNNNNNNNNNNNNNNNNNNNNNNNNNNNNNNNNNNNNNGTTCACTGCCGTATAGGC
AGNNNNNNNNNNNNNNNNNNNNGTTTCAGAGCTATGCTGGAAACAGCATAGCAAGTTGAAATAAGGCTAG
TCCGTTATCAACTTGAAAAAGTGGCACCGAGTCGGTGCGTTCACTGCCGTATAGGCAGNNNNNNNNNNN
NNNNNNNNN**GTTT**CAGAGCTATGCTGGAAACAGCATAGCAAGTTGAAATAAGGCTAGTCCGTTATCAACTT
GAAAAAGTGGCACCGAGTCGGTGCGTTCACTGCCGTATAGGCAGTTTTTTTTGATATCTCCGGGGCTAATT
GAATATGAAGATGAAGATGAAATATTTGGTGTGTCAAATAAAAAGCTGGTGTGCTTAAGTTTGTGTTTTTTTC
TTGGCTTGTTGTGTTATGAATTTGTGGCTTTTTCTAATATTAAATGAATGTAAGATCTCATTATAATGAATAAAC
AAATGTTTCTATAATCCATTGTGAATGTTTTGTTGGATCTCTTCTGCAGCATATAACTACTGTATGTGCTATGG
TATGGACTATGGAATATGATTAAAGATAA

BsaI-based Goden Gate cloning:

pG3H-4A92-dV3RS + p2xPE3RSΔE-P1 + p2xPE3RSΔE-P2 + p2xPE3RSΔE-P3 = 4A92-2xPE3RSΔE (4A92ΔE)

Supplemental Sequence 31. Sequences of primers

Name	Sequence
SPL14-IDF	ATTTCTCCTACCCAAGGGTTCCAAG
SPL14-IDR	GCCTCTACAGAGACCAATCCATCGT

Name	Sequence
SPL14-F	GGAGTGAGTACGGTGTGCGCCCAAATCTCCCTCCA
SPL14-R	GAGTTGGATGCTGGATGGCCTGGTTGTGGCTGGCA
SLR1-IDF	AACCTATCCCAAAGCCGAAACC
SLR1-IDR	TGACAGTGGACGAGGTGGAAGC
SLR1-F	GGAGTGAGTACGGTGTGCGCGCGAGTACCAAGAA
SLR1-R	GAGTTGGATGCTGGATGGTGGCCATCTCCAGCTG
NR2-IDF	TCATGTCGCAGTACCTGGACTACCT
NR2-IDR	GTTTCAGAAGACGAGGCAGGACTTG
NR2-F	GGAGTGAGTACGGTGTGCTCACGCCGGTGTACCA
NR2-R	GAGTTGGATGCTGGATGGGGTCGATCTCCTCCCG
NRT1.1B-IDF	TTGTTAGTCGGACCACTAAGCAAAT
NRT1.1B-IDR	AGGAGGATGGAGGCGATGAG
NRT1.1B-F	GGAGTGAGTACGGTGTGCGACCATGCGGCGATCAT
NRT1.1B-R	GAGTTGGATGCTGGATGGTGGTCATCTGGGCGTGG
AK-IDF	CACATCAGTACCATTCAACTCTCGG
AK-IDR	TGCCACCTTTAGAGAATGAGCAGTC
AK-F	GGAGTGAGTACGGTGTGCTTCTGATCGCTCTCTGTATG
AK-R	GAGTTGGATGCTGGATGGGTTTTACCTTGAAAGAATGT
DHDPS-IDF	ATAGAAGGTGGTGCTGAAGGTGTAA
DHDPS-IDR	TAAGGTTGCTGGCTACAGAAATGAC
DHDPS-F	GGAGTGAGTACGGTGTGCTAACTGCTTTGGCACTAA
DHDPS-R	GAGTTGGATGCTGGATGGGAGAACAGCCTCAAAATG
TubA2-IDF	CGCTATGTACTCCAGTTATTTGCTG
TubA2-IDR	CACCCTCATCACCCTCGTCAC
TubA2-F	GGAGTGAGTACGGTGTGCCCTCACTGACTGCCTCCC
TubA2-R	GAGTTGGATGCTGGATGGATCTCCGCCACGGAGAGCT
Cold1-IDF	TGACTTCACGACTGGACTACCTTGT
Cold1-IDR	AGTCAAGCCACAAAGGGTAAACATC
Cold1-F	GGAGTGAGTACGGTGTGCACAGTGTGCTTTGATTTCAG
Cold1-R	GAGTTGGATGCTGGATGGATCCTCTCCATCTCCATTTT
EPSPS-IDF	GAAACTGATGCCTGCTTACATTGCT
EPSPS-IDR	CAACCCGACAACCAAGTCACCA
EPSPS-F	GGAGTGAGTACGGTGTGCCTGTAGTCGTTGGCTGTG
EPSPS-R	GAGTTGGATGCTGGATGGCCCCATGAATTCCATACAT
ALS-W548-IDF	CTAACCCAGGTGTCACAGTTGTT
ALS-W548-IDR	AGAGCACATACAAACATCATAGGC
ALS-W548-F	GGAGTGAGTACGGTGTGCTTCAGGAGCTGGCATTGAT
ALS-W548-R	GAGTTGGATGCTGGATGGCGGGTTGCCCAAGTATGTA
ACC-IDF	AGTGAAATCTTGCTTCCGTGTTG
ACC-IDR	AATGTAGGCAGGAACATAACTGAGC
ACC-F	GGAGTGAGTACGGTGTGCAGTGGTGAAATTAGGTGGG
ACC-R	GAGTTGGATGCTGGATGGCCAACAGTTCTTCCAGTCAC
ALS-P171-IDF	GGGGCAACCAACCTCGTGTC

Name	Sequence
ALS-P171-IDR	TGCGATGTACCCTGGTAGATTC
ALS-P171-F	GGAGTGAGTACGGTGTGCCAACCAACCTCGTGTCCG
ALS-P171-R	GAGTTGGATGCTGGATGGTTGTGCTTGGTGATGGAG
Xa5-IDF	GGAATTTGCTCGCGTTCGTTCGTTA
Xa5-IDR	GGTTCTCCAAGGCTTCCGTCAT
Xa5-F	GGAGTGAGTACGGTGTGCATGGTCTCCAGCGGCA
Xa5-R	GAGTTGGATGCTGGATGGTCCAAGGCTTCCGTCA
Xa23-IDF	CCTCCCTGTCCACATGAGCTAAACG
Xa23-IDR	ATGGAGCCAAACAGGTAGAACAGCA
Xa23-F	GGAGTGAGTACGGTGTGCAATTCGTCGTGGTGGTGA
Xa23-R	GAGTTGGATGCTGGATGGTGTATACCGGCTACGGCT
ROS-IDF	GAAAATTCTCAATCAGGATACAGCCAAC
ROS-IDR	GCATGCACATATGACTAGAGTTATGTCAAC
ROS-F	GGAGTGAGTACGGTGTGCCACGGAGTTTCTTCCAACT
ROS-R	GAGTTGGATGCTGGATGGCCTTCACCTCTGCTTGTCT
TT1-IDF	AGATTAATGACTAGGATGGTCGAC
TT1-IDR	ACAGTTAGAAAGCAGAAACACCGAT
TT1-F	GGAGTGAGTACGGTGTGCTTCATGTGCTTACCTTG
TT1-R	GAGTTGGATGCTGGATGGTAGGATAGCACTGTAAAAC

Supplemental Sequence 32. Sequences related to pegRNAs and nicking sgRNAs

Target	Mutations	Name	Sequence
OsSPL14	L292I	target	GGTAGTAGTATCCCATGGCTGGG
		rtT/PBS	TGTGCTaTCTCgCTgCTGTCAACgCAGC/CATGGGATACTAC
		nicking	GGTGGATGTCTCGCAGGGGTCGG
OsSLR1	84-bp deletion	target	TATGGGGTCGTGCAAGGACAAGG
		rtT/PBS	TTCTGCGCGACGTCGGCCAT/GTCCTTGCACGACCC
		nicking	CTCCAGCTTCTGCGCGACGTCGG
OsNR2	W779R	target	GGTGATCCAGGCCGTGCTCTGGG
		rtT/PBS	GTCGGGCTGGTCgCgGA/GCACGGCCTGGAT
		nicking	GGAGCATGTCGTCCTCCGTCCGG
OsNRT1.1B	T327M	target	CAGGTCGGCGGCGGAGTCGCCGG
		rtT/PBS	TTGCTCGCCaTCGCaGGCG/ACTCCGCCGCCGA
		nicking	TGATGGTGGTCGCCCAGATGGGG
OsAK	S449L	target	AGGGATATCTGTAGATTGTGTGG
		rtT/PBS	TAACTTCgagAGTAGCgACgC/AATCTACAGATAT
		nicking	ACTAGTTTGGTCAGAAGTACCGG
OsDHDPS	S50N	target	ATTAAAGTGGTCGGCAACACAGG
		rtT/PBS	TTGTTGAGTTgtTTCCTGTa/TTGCCGACCACTT
		nicking	GGAGATCAACCCTTCGACGGAGG
OsTubA2	M268T	target	CGAGATCACCGGGGCGTAGGAGG

Target	Mutations	Name	Sequence
		rtT/PBS	ACTTCAcGCTgTCgTCgT/ACGCCCCGGTGAT
		nicking	GTTCGATGGTGCTCTGAATGTGG
OsCold1	K187T	target	TTACAGGGAAATTGATGAAAAGG
		rtT/PBS	AATGTTTTGATGTCagTcT/CATCAATTTCCCT
		nicking	CTTAGCGATGCATGTCTCCATGG
OsEPSPS	TAP-IVS	target	GCAGTCACGGCTGCTGTCAATGG
		rtT/PBS/Linker	TGGAAtTGtAATGCGAtCATTG/ACAGCAGCCGTGA/AGAAATAT
		nicking	TGTTGAGAAGGATGCGAAAGAGG
OsALS	W548M	target	TTTGGGTATGGTGGTGCAATGGG
		rtT/PBS/Linker	AACCTATCCTCCatcT/GCACCACCATACC/ATTCTACG
		nicking	ACACCATCACCTTCACAGGGAGG
OsALS	P171F	target	CGTCCCGATGGTCGCCATCACGG
		rtT/PBS/Linker	GGCGGaaGACCTGGCCaGTGA/TGGCGACCATCGG/AAATTAGT
		nicking	GACTATGGGCGTCTCCTGGAAGG
OsACC	I1879V	target	CAAGGAAGATGGACTTGGTGTGG
		rtT/PBS/Linker	ACTTCCATGcAcATTCTCgACAC/CAAGTCCATCTTC/AAATTTAC
		nicking	ATATGCCCTAGAATAAGCACTGG
OsXa5	V39E	target	AAGTAGATACCTTATCAAACTGG
		rtT/PBS/Linker	GCCATTCAAGTTCTTGAGCAGTT/TGATAAGGTATC/AGGAATAA
		nicking	AGTTCTTGagCAGTTTGATAAGG
OsXa23	30-bp insertion	target	AGTAGCTGATGTTAGTGAGGCGG
		rtT/PBS/Linker	GCCTTCCTTCCGCCT/TATATAAACCCCCTCCAACCAGGTGCTAAG/CACTAACATCA/AAATTCAT
		nicking	TTCCTTGTTGCATCATCTCAAGG
OsROS	D1425N	target	AGAAAGATCCCAGAATGCTAAGG
		rtT/PBS	AATCAATTGAGTtCTTAG/CATTCTGGGATCT
		nicking	TCCATCGACTGGAACTTTGGGGG
OsTT1	R99H	target	CAGACTTCCGTGTTTTAGTGAGG
		rtT/PBS	TTGTACAGgtGATAgTACTGTTGTGCTTGCTTgCGACTcTTgCgCAC/TAAAACACGGAAG
		nicking	AGAAAGCAGAAACACCGATTCGG

Supplemental References

Cao, Z., Sun, W., Qiao, D., Wang, J., Li, S., Liu, X., Xin, C., Lu, Y., Gul, S.L., Wang, X.C. and Chen, Q.J. (2024) PE6c greatly enhances prime editing in transgenic rice plants. *J Integr Plant Biol* **66**, 1864-1870.

Jiang, Y., Chai, Y., Qiao, D., Wang, J., Xin, C., Sun, W., Cao, Z., Zhang, Y., Zhou, Y., Wang, X.C. and Chen, Q.J. (2022) Optimized prime editing efficiently generates glyphosate-resistant rice plants carrying

homozygous TAP-IVS mutation in EPSPS. *Mol Plant* **15**, 1646-1649.

Liu, Q., Wang, C., Jiao, X., Zhang, H., Song, L., Li, Y., Gao, C. and Wang, K. (2019) Hi-TOM: a platform for high-throughput tracking of mutations induced by CRISPR/Cas systems. *Sci China Life Sci* **62**, 1-7.

Zhang, Q., Zhang, Y., Lu, M.H., Chai, Y.P., Jiang, Y.Y., Zhou, Y., Wang, X.C. and Chen, Q.J. (2019) A novel ternary vector system united with morphogenic genes enhances CRISPR/Cas delivery in maize. *Plant Physiol.* **181**, 1441-1448.
